# Study on the impact of Hfq regulation on spoilage odor formation by *Pantoea agglomerans* inoculated onto chilled chicken meat

**DOI:** 10.1016/j.fochx.2026.104052

**Published:** 2026-05-30

**Authors:** Xiaochen Wang, Yuhang Ma, Yafeng Wang, Lianxin Qi, Yangyong Lv, Shan Wei, Fengguang Zhao, Yingying Wang, Shuaibing Zhang, Yang Lei, Yuansen Hu, Ming Huang

**Affiliations:** a College of Biological Engineering, Henan University of Technology, Zhengzhou 450001, China; b College of Food Science and Technology, Nanjing Agricultural University, Nanjing 210095, China

**Keywords:** Chilled chicken meat, *Pantoea agglomerans*, Hfq, Volatile compounds, HS-GC-IMS

## Abstract

To investigate the impact of *Pantoea agglomerans* on chicken meat spoilage and the regulatory role of *Hfq*, wild-type and Δ*Hfq* strains were inoculated onto chilled chicken meat. Microbial and physicochemical properties were monitored at 4 °C over 0, 1, 3, 5, and 7 days. Parameters assessed included microbial analysis, pH, total volatile basic nitrogen, thiobarbituric acid reactive substances, texture, electronic nose and electronic tongue. Additionally, spoilage compounds were analyzed using headspace-gas chromatography-ion mobility spectrometry and headspace-solid-phase microextraction-gas chromatography–mass spectrometry. Partial correlations between these indicators were analyzed. The results showed that the WT strain exhibited faster proliferation during the middle storage stage and induced greater increases in pH, TVB-N, TBARS, and spoilage-related compounds, alongside more severe texture degradation and stronger spoilage odors. In contrast, the Δ*Hfq* strain caused milder changes. The findings suggest that *P. agglomerans* is linked to accelerated spoilage, with Hfq potentially correlating with this process under the present model.

## Highlights

• *Hfq* deletion correlates with weaker protein breakdown by *P. agglomerans* in chicken.
• A total of 71 unique VOCs were identified by GC-IMS and GC–MS after duplicate removal.
• The sec-butylamine was detected in chicken contaminated with *P. agglomerans* on 7 days.

## Graphical abstract

**Figure:**
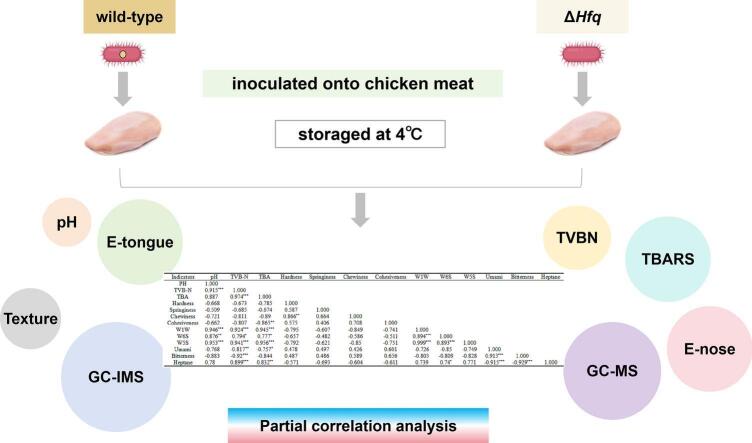

## 1 Introduction

Chicken meat is one of the most widely consumed types of meat globally, valued for its nutritional benefits and ease of processing and storage. However, its high protein content, moisture, and fine muscle fiber structure make it particularly vulnerable to microbial contamination and spoilage (Marmion et al., 2021). *Pantoea*, a group of Gram-negative bacteria, are significant spoilage organisms due to their adaptability to low temperatures and their ability to hydrolyze proteins and fats. Among these, *P. agglomerans* has been identified in refrigerated meat, where it causes considerable spoilage, including acidification and degradation of texture, thus shortening the shelf life of products (Casaburi et al., 2015; Lei et al., 2022b). This bacterium can thrive in a range of environments, tolerating pH levels between 4.5 and 9 and temperatures from 4 °C to 40 °C. The spoilage effect of *P. agglomerans* is primarily driven by its production of extracellular proteases and lipases, which degrades proteins and fats in chicken meat, leading to acidification and gas production (Walterson et al., 2015). As a dominant spoilage bacterium, *P. agglomerans* not only flourishes on chicken meat but also influenced by intracellular regulatory factors that contribute to spoilage.

Hfq is a global regulatory factor that plays a crucial role in the post-transcriptional regulation of bacteria metabolism. It influences a variety of physiological and biochemical processes, including growth, spoilage potential, motility, environmental adaptability, and toxin production (Pusic et al., 2018). In studies on *Shewanella baltica*, *Hfq* mutants showed reduced growth during the exponential phase and a decreased stationary phase population. However, no significant growth defects were observed in *Hfq* mutants of *Listeria monocytogenes* and *Staphylococcus aureus* (Chao et al., 2010). These findings suggest that the regulatory impact of Hfq varies across different bacterial species.

Current research on refrigerated chicken primarily focuses on the evolution of microbial communities, often relying exclusively on bacterial counts. This approach overlooks the specific spoilage capabilities of these microorganisms involved. To address this gap, our study aims to investigate the regulatory factors that influence the spoilage caused by *P. agglomerans* in chicken meat. By examining both wild-type (WT) and Δ*Hfq* strains in situ, we seek to provide valuable insights into how Hfq regulation affects spoilage processes in chilled chicken. Through comprehensive analysis, we evaluate accelerated spoilage correlated with *P. agglomerans* and alterations potentially associated with *Hfq* deficiency. This research lays the foundation for future advancements in meat preservation technology and quality control.

## 2 Materials and methods

### 2.1 Sample preparation

The wild-type *P. agglomerans* strain (HJS002) was isolated from chicken meat, and its complete genome sequence is available in GenBank (accession numbers: cp090207.1-cp090211.1). The Δ*Hfq* strain was previously developed in our laboratory (detailed information on the *Hfq* gene deletion can be found in the supplementary data). Fresh chicken breast samples were obtained from the local Shuanghui fresh meat supermarket. The samples were rinsed twice with sterile water to remove surface bacteria, followed by UV-C irradiation for 0.5 h (Ma, 2022). Previous studies have shown that UV-C treatment, at appropriate doses, effectively inactivates microorganisms like *Salmonella* on chicken surfaces without affecting meat quality (García-Gimeno et al., 2024). A UV-C treated inoculation model was thus constructed to eliminate interference from indigenous microorganisms. Next, the chicken samples were inoculated with suspensions of either the WT or Δ*Hfq* strains. The actual initial inoculum was confirmed by plate counting 3.09 ± 0.19 lg CFU/g for the WT strain and 3.18 ± 0.09 lg CFU/g for the Δ*Hfq* strain. Non-inoculated samples served as the control group. The samples were placed in sterile petri dishes and packaged in a tray configuration using PVC film, with an oxygen transmission rate of 5000 cm^3^/(m^2^·24 h·atm) at 23 °C and 75% relative humidity (estimated to be approximately 1600 cm^3^/(m^2^·24 h·atm) at 4 °C based on the temperature dependence of gas permeability in PVC film). All samples were stored at 4 °C and aseptically sampled at days 0, 1, 3, 5, and 7. Each experiment included three biological replicates, using independent chicken breast samples from different batches. Separate samples were analyzed at each time point to avoid interference from repeated sampling.

### 2.2 Microbiological analysis

Under aseptic conditions, 25 g of chicken meat sample was placed in a stomacher bag, mixed with 225 mL of 0.85% sterile physiological saline, and homogenized at room temperature for 10 min. Ten-fold serial dilutions were prepared for each sample, and 0.1 mL aliquots from appropriate dilutions was spread-plated onto the respective agar plates. Total viable counts (TVC) were enumerated on Tryptic Soy Agar (TSA) after incubation at 30 °C for 1 day. *Pseudomonas* spp. were enumerated on *Pseudomonas* CFC Selective Agar after incubation at 25 °C for 2 days, and lactic acid bacteria (LAB) were enumerated on de Man Rogosa Sharpe (MRS) agar after incubation at 25 °C for 5 days. All analyses were performed in triplicate for each sample (Balamatsia et al., 2006). To verify that the inoculated *P. agglomerans* WT and Δ*Hfq* strains were free from contamination during storage, three single colonies were randomly picked from the TSA plates of each group (WT and Δ*Hfq*) at days 0, 1, 3, 5, and 7. These colonies were subjected to 16S rRNA gene amplification and sequencing.

### 2.3 pH measurement

To measure the pH, 10 g of the sample were homogenized with 100 mL of distilled water and filtered. The pH was then determined using a calibrated pH meter, with three replicates for each sample (Dong et al., 2025).

### 2.4 Total volatile basic nitrogen (TVB-N)

The TVB-N content was meaured using an automatic Kjeldahl nitrogen analyzer, following the GB 5009.228–2016 standard (Determination of Volatile Basic Nitrogen in Food). A 10 g sample was immersed in 75 mL of distilled water for 30 min, followed by the addition of 1 g of MgO. The sample was connected to a distillation apparatus for nitrogen analysis, and a blank test was also conducted. All analyses were performed in triplicate.

### 2.5 Thiobarbituric acid reactive substances (TBARS) analysis

The TBARS value was determined using the method described by Cai et al. (2021). A 5 g meat sample was homogenized with 25 mL of 7.5% trichloroacetic acid. The mixture was centrifuged at 3600 ×*g* for 20 min, and 5 mL of the supernatant was combined with an equal volume of 0.02 mol/L TBA solution. The mixture was heated in boiling water for 30 min. After cooling to room temperature, the mixture was centrifuged again at 1600 rpm for 5 min. The supernatant was collected and mixed with 5 mL of chloroform, and the absorbance was measured at 532 nm.

### 2.6 Texture evaluation

Chicken samples were cut into 2 cm × 2 cm pieces and analyzed for texture using a texture analyzer. A cylindrical probe (P50) was used, with pre-test, test, and post-test speeds set to 2 mm/s. The deformation was set to 40%, and the test duration was 5 s. Parameters measured included hardness, elasticity, and chewiness (Zhou et al., 2014).

### 2.7 Electronic nose analysis

A 2 g sample was homogenized into a paste and placed into a 50 mL centrifuge tube. Each sample was tested in triplicate, sealed with a film, and incubated at 60 °C for 30 min before analysis using a PEN3 electronic nose (Airsense, Germany), which is equipped with 10 sensors (Table 1). The direct headspace injection method was employed, with the following sampling conditions: 1 s per group for sampling, 60 s of sensor self-cleaning, 5 s of sample injection time, an injection flow rate of 400 mL·min^−1^, and a total analysis time of 80 s (Chen et al., 2022). At 69–71 s, the *E*-nose sensors reached dynamic equilibrium, exhibiting a stable signal plateau with minimal fluctuation and optimal repeatability. Data from this interval were used for analysis, providing an objectively representation of the sample's true volatile characteristics.

**Table 1:** Performance Description of Electronic Nose Sensors.

Array Number	Name	Performance Description
1	W1C	Aromatic ingredients, benzene
2	W5S	High sensitivity, very sensitive to nitrogen oxides
3	W3C	Aromatic component sensitivity, ammonia
4	W6S	Selective mainly for hydrides
5	W5C	Short-chain alkane aromatic components
6	W1S	Sensitive to methyl groups
7	W1W	Sensitive to sulffdes
8	W2S	Sensitive to aldehydes and ketones
9	W2W	Aromatic components, sensitive to organic sulfides
10	W3S	Sensitive to long chain alkanes

### 2.8 Electronic tongue analysis

A 20 g sample was homogenized with 200 mL of water, then centrifuged and filtered before analysis. Sensory properties were assessed using an SA402B electronic tongue (Insent, Japan), which is equipped with 8 sensors: C00 (bitterness), AE1 (astringency), CA0 (sourness), CT0 (saltiness), AAE (umami), GL1 (sweetness), ANO (b-bitterness2), and BTO (h-bitterness). Prior to testing, the sensors were cleaned sequentially with negative and positive electrode cleaning solutions for 90 s each, washed twice with reference solution, and then zeroed at the equilibrium position for 30 s to complete calibration and drift correction. The umami measurement was performed for 30 s, followed by rinsing with reference solution and aftertaste testing for 30 s. Each test was repeated four times, with the first cycle discarded, and the average of the last three cycles taken as the result (Bao et al., 2022).

### 2.9 HS-GC-IMS analysis

Volatile components were analyzed using the Flavor Spec1H1–00053 GC-IMS system (G.A.S, Dortmund, Germany), following a modified method from Yang et al. (2022). A 2 g sample was placed into a 20 mL headspace vial and incubated at 60 °C for 20 min. The headspace sampling conditions were as fllows: incubation temperature at 60 °C, incubation time of 20 min, shaking speed of 500 rpm, injection needle temperature at 85 °C, and injection volume of 500 μL. Prior to sample analysis, vial blank experiments were performed, and no substances were detected in the blanks. The GC-IMS analysis conditions were: analysis time of 35 min, chromatographic column type WAX, 15 mID × 0.53 mm, column temperature at 60 °C, carrier gas/Drift gas N_2_, and IMS temperature at 45 °C. Under these chromatographic conditions, the retention index (RI) was calculated using a series of C₄ ∼ C₉ n-alkanones (2-butanone, 2-pentanone, 2-hexanone, 2-heptanone, 2-octanone, 2-nonanone), with matching thresholds set at RI ± 30 and drift time (Dt) ± 0.02. VOCs were identified by comparing their retention and drift times with the built-in NIST database and IMS database of the software.

### 2.10 HS-SPME-GC–MS analysis

Volatile compounds were detected using a GC–MS system (7890B—7000C, Agilent Technologies, USA). Solid-phase microextraction (SPME) was performed using a manual SPME injector (Supelco, USA) with a 50/30 μm PDMS/CAR/DVB (2 cm) extraction fiber. Following a modified method of Tan et al. (2021), 3 g of sample was placed in a 20 mL extraction vial, and 10 μL of 50 μg/mL 2-methyl-3-heptanone internal standard was added before quick sealing. The SPME extraction fiber was conditioned by inserting it into the injector at 250 °C until no background peaks were observed. Procedural blank experiments were performed prior to volatile organic compound analysis. All volatile organic compounds detected in blanks were regarded as potential contaminants and excluded directly from subsequent data analysis without peak area subtraction or ratio-based correction, to minimize background interference. The fiber was then inserted into the headspace vial and heated for 30 min for extraction. Afterward, it was placed into the GC injection port at 250 °C for 3 min of desorption. GC conditions included a DB-5MS column (30 m × 250 μm, 0.25 μm film thickness), injection port temperature at 250 °C, helium as the carrier gas, and a flow rate of 1.0 mL/min. The temperature program started at 40 °C for 3 min, then ramped at 6 °C/min to 100 °C, followed by a 10 °C/min ramp to 230 °C, with a 3 min hold. The retention index (RI) was calculated using n-alkane standard, and the RI values of some compounds were compared with those from the online database (https://webbook.nist.gov) for additional identification. Mass spectrometry conditions included electron impact (EI) ionization, ion source temperature at 230 °C, transfer line temperature at 230 °C, electron energy at 70 eV, and a mass scan range of 40 u to 550 u. VOCs were putatively identified by comparing their mass spectra with the NIST 17 database (match factor ≥ 80%) and retention indices reported in the literature. Semi-quantitative analysis was performed to quantify VOCs, using 2-methyl-3-heptanone as the internal standard. Relative semi-quantitative equivalents of each volatile compound, expressed as internal-standard equivalents in μg/kg, were determined by comparing the peak area ratio of each compound to that of the internal standard.

### 2.11 Statistical analysis

All experiments were conducted with three biological replicates, and the data are presented as means ± standard deviations (SD). Two-way ANOVA (fixed factors: group × storage time) and Duncan’s multiple range test were employed separately for different indicators. Although this test is relatively liberal, it is suitable for exploratory comparisons among the limited treatment groups in this work. All statistical analyses were implemented using SPSS 25.0, and differences were regarded as statistically significant at *p* < 0.05.

## 3 Results and discussion

### 3.1 Microbial analysis

**Fig. 1:**
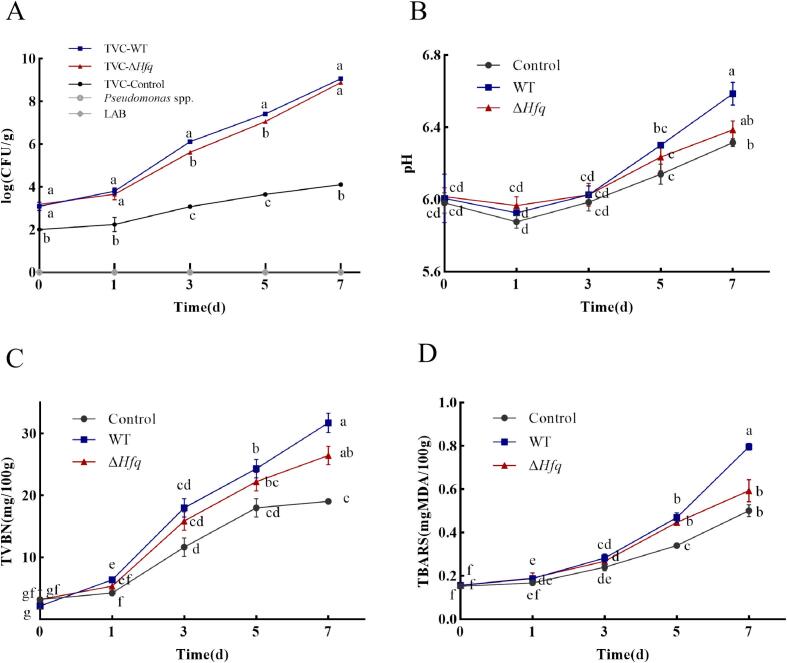
Microbial growth (A), pH changes (B), TVB-N (C) and TBA (D) of chicken breast samples inoculated with WT strain, the Δ*Hfq* strain of *P. agglomerans*, and a non-inoculated Control group at 4 °C for seven days. Note: Different lowercase letters denote significant differences at different times during storage (*p* < 0.05).

The results of the microbiological analysis are presented in Fig. 1A. On day 0 of storage, the initial colony counts of the WT and Δ*Hfq* groups were 3.09 and 3.18 lg CFU/g, respectively, with no significant difference in the initial bacterial load between the two groups. Over the storage period, the total viable counts of both the WT and Δ*Hfq* groups were significantly higher than those in the control group. On day 3 and day 5 of storage, colony counts in the Δ*Hfq* group were significantly lower by 0.5 and 0.36 log units, respectively, compared to the WT group. However, there was no significant difference in total viable counts between the two groups at the early stage (1 d) and late stage (7 d) of storage. In the present UV-C treated inoculation model, the differences in the proliferation rate of *P. agglomerans* during the middle storage stage may be associated with *Hfq* gene deletion, while its final growth yield was not significantly affected. Throughout the storage period, counts of *Pseudomonas* spp. and lactic acid bacteria in all treatment groups were below the limit of detection (LOD = 2.00 lg CFU/g), eliminating interference from other bacterial species in the experimental results. To verify the purity of the inoculated strains, three single colonies were randomly selected from TSA plates of the WT and Δ*Hfq* groups on days 0, 1, 3, 5, and 7 of storage, respectively, and analyzed via 16S rRNA sequencing and NCBI BLAST alignment (supplementary data). The 16S rRNA sequencing confirmed the identity of picked colonies as *P. agglomerans* at the species level, with 100% query coverage and 99.05%–99.06% identity for all samples (Table S1). Notably, this species-level identification cannot provide strain-specific quantitative information on the inoculated strains over storage time. Collectively, these data support that no detectable target-group contamination occurred in the system, which helps guarantee the reliability of subsequent experimental results.

### 3.2 pH changes

The pH values of the chicken meat samples throughout the storage period are shown in Fig. 1B. The initial pH of all samples was 6.0. During the early stages of storage, the pH decreased, likely due to glycolysis in muscle tissue post-slaughter, which produces lactic acid and other acidic compounds (Cai et al., 2021). As storage continued, protein degradation resulted in the formation of amines and other alkaline compounds, leading to a gradual increase in pH (Wang et al., 2023). For the first five days, no significant difference in pH was observed between the WT and Δ*Hfq* groups (*p* > 0.05), suggesting that bacterial protein degradation had a minimal impact on pH during this period. However, on day 7, the pH of the WT group increased to 6.58, whereas the Δ*Hfq* group's pH was 6.38, showing a significant difference (*p* < 0.05). Under the present UV-treated in situ model, the observed pH difference may be associated with *Hfq* gene deletion, suggesting that *Hfq* deletion could possibly be linked to changes in the metabolic pathways of *P. agglomerans*, which may affect the expression or activity of proteins involved in protein degradation, and ultimately possibly lead to accelerated protein degradation and a faster pH increase in the WT strain.

### 3.3 TVBN changes

Total volatile basic nitrogen (TVB-N) is a key indicator of meat freshness, reflecting the degradation of proteins into ammonia and amines due to the influence of endogenous enzymes and bacterial activity (Liu et al., 2022). According to the GB 2707–2016 standard for fresh poultry, the TVB-N content in chicken meat should not exceed 15 mg/100 g. As shown in Fig. 1C, the TVB-N values of all samples increased over the storage period. Initially, TVB-N values ranged from 2.11 to 3.17 mg/100 g. On day 3, the WT group reached 17.97 mg/100 g, while the Δ*Hfq* group reached 15.85 mg/100 g, both exceeding the standard for fresh meat. By day 5, the control group also surpassed this limit, indicating that the inoculation of *P. agglomerans* reduced the storage life of chicken meat by approximately two days. On day 7, the TVB-N value of the WT group reached 31.72 mg/100 g, while the Δ*Hfq* group had a lower value of 26.43 mg/100 g. From day 1, the TVB-N values of the Δ*Hfq* group were consistently lower than those of the WT group, with the control group consistently exhibiting the lowest values throughout. This suggests that inoculation with *P. agglomerans* is associated with accelerated protein degradation, correlating with faster spoilage under the present UV-treated in situ model. Furthermore, the lower TVB-N production in the Δ*Hfq* group may be associated with the absence of *Hfq*, which could contribute to reduced formation of nitrogenous volatile compounds. Previous studies have shown that Hfq inhibits ornithine decarboxylase activity in *Yersinia enterocolitica*, reducing the conversion of ornithine to putrescine (Kakoschke et al., 2014).

### 3.4 TBA analysis

The TBA value is an critical indicator of lipid oxidation in meat, resulting from the degradation of unsaturated fatty acids and phospholipids (Domínguez et al., 2019). As illustrated in Fig. 1D, the TBA values of chicken samples stored at 4 °C increased over time. During the first three days, no significant differences in TBA values were observed between samples (*p* > 0.05). However, as storage continued, TBA values significantly increased across all groups. The WT group exhibited the largest increase, reaching 0.79 mg MDA/100 g by day 7, which was significantly higher than the Δ*Hfq* group (0.59 mg MDA/100 g, *p* < 0.05). Although the control group also showed an increase, its TBA values remained lower than those of the inoculated groups. To assess the impact of UV pretreatment on the initial lipid oxidation, TBA values of UV treated and untreated samples stored at 4 °C for 0  and 1 days were compared. No significant difference was found between the two groups (Table S2). These results suggest that bacterial is associated with accelerated lipid oxidation, a process likely correlated with Hfq under the present UV-treated in situ model. *Hfq* deletion is likely associated with altered lipase expression and lipid metabolism in *P. agglomerans* during chicken meat spoilage. Previous studies have shown that in *Pseudomonas*, Hfq binds to Rsmy, enhancing the stability of *lipA* mRNA, which in turn promotes lipase synthesis (Liu et al., 2017b). Lipase is crucial in fat metabolism, and its increased activity accelerates the oxidation of unsaturated fatty acids, leading to the formation of secondary products such as malondialdehyde, thereby increasing TBA values.

### 3.5 Texture profile analysis

Table 2 presents the changes in texture properties (hardness, springiness, chewiness, and cohesiveness) of chicken under different treatments stored at 4 °C. Over time, all groups showed significant decreases in these properties (*p* < 0.05). By day 7, the hardness of the WT and Δ*Hfq* groups had decreased by 53.6% and 41.6%, respectively, while springiness decreased by 29.3% and 26.7%. These changes were likely attributed autolytic enzymes accelerating protein denaturation and disrupting the connective tissue structure, with microbial activity further degrading muscle fibers, leading to looser protein structures and reduced hardness and springiness (Viji et al., 2015). Lou et al. (2023a) observed similar findings, noting that *Shewanella baltica* produce Lon protease during growth, resulting in reduced fish hardness. In *Dickeya dadantii*, Hfq stabilizes mRNA for protease genes, and its deletion reduces extracellular protease activity by 50% (Leonard et al., 2021). The present study suggests that the decrease in hardness in the non-inoculated group was primarily due to enzyme-driven autolysis. It is hypothesized that extracellular proteases secreted by *P. agglomerans* may contribute to accelerated protein hydrolysis and subsequent deterioration of meat texture under the present UV-treated in situ model. Although chewiness and cohesiveness also significantly decreased by day 7 (*p* < 0.05), no significant differences in these properties were observed between groups during the first five days (*p* > 0.05). This suggests that early spoilage was mainly influenced by enzyme-driven autolysis, with minimal influence from *P. agglomerans*. These findings align with those of Lou et al. (2023b), who reported similar trends in texture degradation in *Scomberomorus* fish inoculated with *S. baltica* over a 10-day storage period.

**Table 2:** Texture evaluation of chicken samples stored at 4 °C for 7 days.

Parameters	Time(d)	Samples		
		Control	WT	Δ*Hfq*
Hardness/g	0	3318 ± 188^Aa^	3563 ± 271^Aa^	3528 ± 295^Aa^
	1	2856 ± 130^ABa^	2851 ± 91^Ba^	2867 ± 237^Ba^
	3	2965 ± 243^Ba^	2955 ± 160^Ba^	2961 ± 85^Ba^
	5	2511 ± 197^Ba^	2016 ± 97^Cb^	2398 ± 131^Ca^
	7	2054 ± 139^Ca^	1538 ± 288^Cb^	1938 ± 189^Dab^
Springiness	0	0.75 ± 0.02^Aa^	0.73 ± 0.02^Aa^	0.72 ± 0.02^Aa^
	1	0.69 ± 0.05^ABa^	0.69 ± 0.05^ABa^	0.70 ± 0.14^ABa^
	3	0.64 ± 0.02^Ba^	0.59 ± 0.03^Ba^	0.59 ± 0.06^ABa^
	5	0.62 ± 0.03^Ba^	0.54 ± 0.01^Ba^	0.58 ± 0.03^Ba^
	7	0.60 ± 0.01^Ba^	0.53 ± 0.01^Bb^	0.55 ± 0.01^Bb^
Chewiness	0	1460.47 ± 87.42^Aa^	1504.99 ± 72.62^Aa^	1531.97 ± 3.28^Aa^
	1	1281.82 ± 67.06^ABa^	1344.21 ± 24.24^Aa^	1299.14 ± 14.58^Ba^
	3	1118.78 ± 64.15^Ba^	1176.05 ± 24.61^Ba^	1143.79 ± 18.50^Ca^
	5	1067.44 ± 15.69^Ba^	968.66 ± 79.33^Ba^	1020.64 ± 12.45^Da^
	7	997.44 ± 33.98^Ba^	748.62 ± 58.41^Cb^	873.51 ± 60.41^Dab^
Cohesiveness	0	0.48 ± 0.007^Aa^	0.50 ± 0.010^Aa^	0.49 ± 0.001^Aa^
	1	0.48 ± 0.014^Aa^	0.48 ± 0.001^Aa^	0.49 ± 0.012^Aa^
	3	0.47 ± 0.007^Aa^	0.47 ± 0.008^Aa^	0.47 ± 0.010^Aa^
	5	0.45 ± 0.025^ABa^	0.44 ± 0.002^Ba^	0.45 ± 0.021^ABa^
	7	0.42 ± 0.003^Ba^	0.39 ± 0.007^Cb^	0.42 ± 0.003^Bab^

Note: Different uppercase letters indicate significant differences in the same column (p < 0.05); different lowercase letters indicate significant differences in the same row (p < 0.05).

### 3.6 *E*-nose analysis

**Fig. 2:**
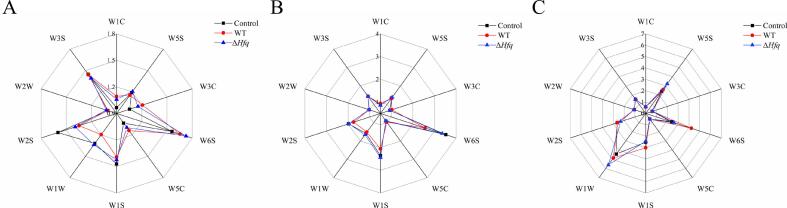
Changes in electronic nose response values of different chicken samples at 4 °C for seven days. (A) Day 1. (B) Day 3. (C) Day 7. Note: WT: Wild-type inoculation group; Δ*Hfq*: *Hfq* deletion mutant inoculation group; Control: Uninoculated control group.

The electronic nose (E-nose) is an instrumental technique widely used to analyze volatile compound profiles in food samples (Bai et al., 2022). Rather than replicating human olfactory perception, it detects sensor response patterns associated with volatiles compounds. In this study, the E-nose we used to evaluate the volatile sensor response patterns of chicken samples inoculated with WT and Δ*Hfq* strains of *P. agglomerans* at 4 °C. As shown in Fig. 2, the E-nose sensor output signal intensities increased over time. By day 7, sensors W1W, W6S, and W5S recorded higher signals, indicating enhanced responses associated with sulfides, hydrides, and nitrogen oxides, respectively. This increase may be associated with the enzymatic activity of *P. agglomerans*, which corresponds with elevated TVB-N and TBA values. Longer storage periods led to more pronounced changes in volatile sensor patterns; however, the Δ*Hfq* strain exhibited lower hydride-related sensor responses, suggesting that the absence of *Hfq* may reduce bacterial growth and enzyme secretion. This observation is consistent with Wang et al. (2022a), who reported a similar correlation between volatile compounds and bacterial metabolism in refrigerated pork.

**Fig. 3:**
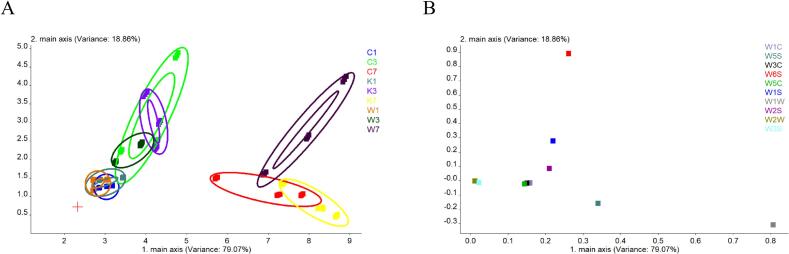
Principal component analysis of electronic nose response data for different chicken samples at 4 °C for seven days. (A) Score plot. (B) Loading plot. Note: C, W, K: non-inoculated, WT-inoculated, Δ*Hfq*-inoculated groups, respectively. 1, 3, 7: storage days, respectively.

To further investigate differences in volatile profiles among the chicken samples, principal component analysis (PCA) was performed. The PCA score plot and loading plots are shown in Fig. 3. PC1 and PC2 accounted for 79.07% and 18.86% of the variance, respectively, with a cumulative contribution of 97.93%, effectively distinguishing the volatile sensor response patterns of the samples. Typically, a total contribution rate above 70% indicates an effective PCA model (Lan et al., 2022), confirming that the *E*-nose reliably captured the differences in volatile profiles. Fig. 3B further shows the relationship between individual sensors and the PCA components, with W1W contributing most to PC1 and W6S having the greatest influence on PC2.

### 3.7 E-tongue analysis

**Fig. 4:**
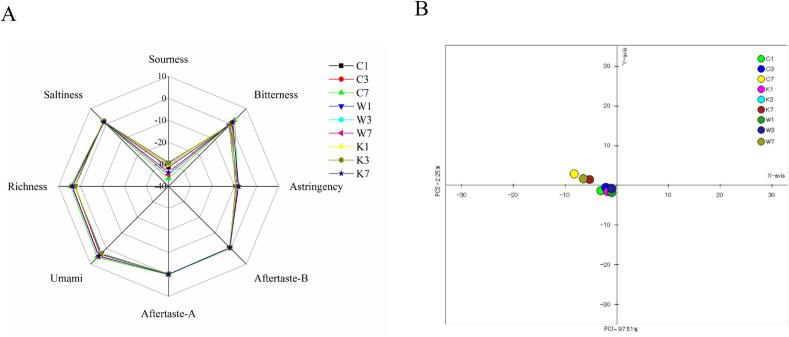
Results of electronic tongue detection for different chicken samples at 4 °C for seven days. (A) Taste indicator radar graph. (B) PCA analysis graph. Note: C, W, K: non-inoculated, WT-inoculated, Δ*Hfq*-inoculated groups, respectively. 1, 3, 7: storage days, respectively.

The electronic tongue (*E*-tongue) is an instrumental technique that detects sensor response patterns associated with taste properties, assessing attributes such as sourness, bitterness, astringency, aftertaste bitterness, aftertaste astringency, umami, richness, and saltiness (Liu et al., 2017a). As shown in Fig. 4A, none of the chicken samples exhibited sensor responses related to sourness, with values below −13, the tasteless point corresponding to the reference solution (30 mM KCl + 0.3 mM tartaric acid), indicating no detectable sourness. In comparison to fresh chicken, samples stored for seven days demonstrated significant differences in sensor response patterns related to taste, particularly in umami, bitterness, richness, and saltiness. As storage time increased, sensor responses associated with bitterness, astringency, aftertaste bitterness, aftertaste astringency, richness and umami were enhanced, while those related to saltiness decreased. Notably, there were significant differences in sensor responses for umami and bitterness between the inoculated and control samples at the end of the storage period (*p* < 0.05). This may be attributed to protease secretion by *P. agglomerans*, which could facilitate the breakdown of muscle proteins into smaller nitrogenous compounds.

PCA based on E-tongue sensor responses (Fig. 4B) revealed that PC1 and PC2 accounted for 97.51% and 2.25% of the variance, respectively, yielding a cumulative contribution of 99.76%. This indicates that PCA effectively differentiates the sensor response patterns related to taste among the various chicken samples.

### 3.8 HS-GC-IMS analysis

**Fig. 5:**
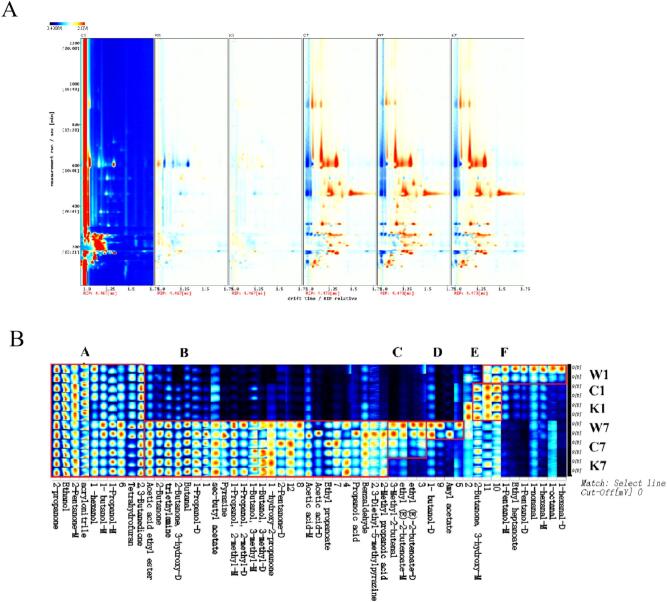
Volatile compounds of different chicken meat identiffed by GC-IMS. (A) two-dimensional differential map. (B) fingerprint. Note: C, W, K: non-inoculated, WT-inoculated, Δ*Hfq*-inoculated groups, respectively. 1, 7: storage days, respectively.

HS-GC-IMS was employed to analyze the VOCs in chicken samples inoculated with WT (W group), Δ*Hfq* (K group), and non-inoculated control (C group) strains at 1 and 7 days of storage. This technique is recognized for its high sensitivity and ease of use, enabling the detection of both monomers and polymers (Sun et al., 2024). A 2D difference plot (Fig. 5A) was generated to visualize the variations in volatile compounds, with the x-axis representing ion migration time and the y-axis showing the gas chromatography retention time. Red vertical lines indicate reaction ion peaks, with each point on either side of the peak representing a volatile compound. The color intensity reflects compound concentration. As shown in the figure, significant differences in both intensity and diversity of volatile compounds were observed among the treatment groups. Qualitative analysis identified 55 signals, including 43 tentatively assigned compounds and 12 unidentified compounds (Table S3), such as aldehydes, alcohols, ketones, esters, acids, and other organic compounds. To evaluate the impact of UV light treatment on VOCs in chicken meat, the study compared VOC profiles in UV-treated and untreated groups at both 0 and 1 days of storage. The results indicated no significant difference in the overall VOC composition between the treated and untreated groups at these storage times (*p* > 0.05). After eliminating substances with large inter-group variations, the similarity of VOCs between treated and untreated groups was greater than 85% (Table S4). PCA results further showed no distinct separation between samples from different treatment groups at the same storage time (Fig. S1), indicating that UV treatment did not significantly affect the overall VOC characteristics during the initial storage period.

The positions of the volatile compounds in the GC-IMS spectra are displayed in Fig. 5B. The volatile substances in different treatment groups at various storage times showed distinct characteristic features and were distributed in different regions. Region A, which represents peaks throughout the entire storage period, included tentatively identified ethanol, 2-pentanone-M, 1-hexanol, and 1-propanol-M, with ethanol showing the highest concentration. Regions B, C, and D mainly corresponded to peaks at 7 days of storage, including tentatively identified acetic acid ethyl ester, 2-butanone, triethylamine, 2-butanone, 3-hydroxy-M, butanal, acetic acid-M, and pyrazine. The WT group exhibited higher levels of tentatively identified amyl acetate, 1-butanol-D, and benzaldehyde compared to the control and Δ*Hfq* groups. Benzaldehyde production, primarily associated with bacterial amino acid metabolism, is known to correlate with meat spoilage (Mansur et al., 2019). Regions E and F represented peaks at 1 day of storage, with the WT group showing higher concentrations of tentatively identified aldehydes, such as 1-hexanal-D, 1-hexanal-M, and 1-octanal, compared to the control group. Tentatively identified aldehydes, as oxidation products of unsaturated fatty acids, contribute distinct odor to chicken meat, although their concentration typically decreases as storage time progresses (Wang et al., 2022b). These changes in compound levels can influence the flavor of the meat, potentially resulting in undesirable odors or tastes. However, GC-IMS provides only tentative identification of individual volatile compounds compared to the more precise GC–MS method (Sun et al., 2023). Therefore, additional GC–MS analysis was conducted to further examine the volatile compounds in the inoculated chicken samples.

### 3.9 HS-SPME-GC–MS analysis

**Fig. 6:**
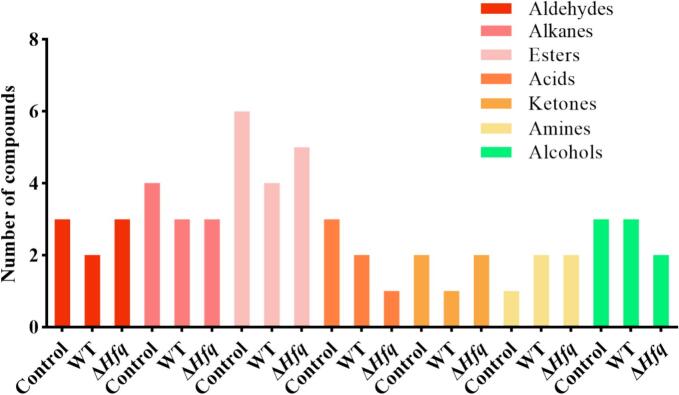
The number of volatile compounds identified in three groups of chicken samples via HS-SPME-GC–MS.

The release of spoilage volatile compounds during meat storage is closely linked to the growth of specific microorganisms (Argyri et al., 2015). In this study, HS-SPME-GC–MS was used to analyze the volatile compounds in chicken samples. VOCs detected in procedural blanks and non-target interfering substances identified in samples were classified as potential contaminants and summarized in Table S5, these substances were excluded from subsequent data analysis and biological interpretation. After exclusion, a total of 30 volatile gases were putatively detected, including 3 aldehydes, 5 alkanes, 8 esters, 4 acids, 3 ketones, 3 amines and 4 alcohols compounds (Table 3). The control group showed 22 compounds, including 3 aldehydes, 4 alkanes, 6 esters, 3 acids, 2 ketones, 1 amine and 3 alcohols compounds. Both the WT and Δ*Hfq* groups displayed 17 and 18 compounds, respectively (WT: 2 aldehydes, 3 alkanes, 4 esters, 2 acids, 1 ketone, 2 amines and 3 alcohols compounds; Δ*Hfq*: 3 aldehydes, 3 alkanes, 5 esters, 1 acid, 2 ketones, 2 amines and 2 alcohols compounds) (Fig. 6). Notably, some compounds, such as hexanoic acid and 5-hepten-2-one, appeared only after 7 days of storage, indicating that storage time significantly influences the volatile profile (Lou et al., 2023b). Compared to fresh chicken, inoculation with *P. agglomerans* resulted in a marked increase in most aldehydes, acids, and alcohols, while the relative semi-quantitative equivalents of alkanes and esters generally decreased. This trend was particularly evident in the WT group. Specifically, the semi-quantitative equivalents of hexanal and nonanal increased by approximately 6 and 1 times, respectively, while 1-pentanol increased by about 21 times. The semi-quantitative equivalent of toluene decreased by 10.68%, and compounds such as pentane, 2,2-dimethyl-, ethanamine, *n*-methyl-, benzeneethanol, α-methyl- were also affected.

**Table 3:** Comparisons of the detected VOCs in inoculated and non-inoculated chicken samples by HS-SPME-GC–MS.

Compounds	Count	RI	Semi-quantitative equivalent (μg/kg)					
			1d			7d		
			C1	W1	K1	C1	W1	K1
Aldehydes								
Hexanal	1	798	3.28 ± 0.36^c^	17.80 ± 2.05^ab^	22.36 ± 2.96^ab^	18.24 ± 1.66^ab^	25.39 ± 2.53^a^	14.41 ± 0.57^b^
Nonanal	2	1101	18.41 ± 0.89^b^	17.71 ± 0.05^b^	21.41 ± 10.97^ab^	24.30 ± 0.58^a^	32.83 ± 2.95^a^	29.02 ± 18.09^ab^
Decanal	3	1128	4.04 ± 1.52^b^	ND	ND	22.57 ± 2.02^a^	ND	14.01 ± 8.29^ab^

Alkanes								
Heptane	4	725	4.61 ± 2.24^a^	4.88 ± 4.53^a^	6.78 ± 1.28^a^	4.13 ± 0.35^a^	3.53 ± 0.35^a^	ND
Toluene	5	768	31.17 ± 1.35^a^	32.80 ± 11.31^a^	47.60 ± 17.42^a^	37.94 ± 1.85^a^	27.84 ± 8.30^a^	31.05 ± 2.41^a^
Undecane	6	1098	4.36 ± 0.14^a^	ND	ND	3.82 ± 0.98^a^	ND	ND
Nonane, 4,5-dimethyl-	7	1058	91.47 ± 6.21	ND	ND	ND	ND	ND
Pentane, 2,2-dimethyl-	8	986	ND	4.06 ± 2.78^a^	12.85 ± 8.34^a^	ND	ND	ND

Esters								
Formic acid, ethenyl ester	9	N.A.	1.43 ± 0.37^a^	ND	ND	9.96 ± 4.64^a^	ND	8.97 ± 2.69^a^
Ethyl Acetate	10	601	44.06 ± 1.36	ND	ND	ND	ND	ND
1,2,4-Benzenetricarboxylic acid, 1,2-dimethyl ester	11	886	3.37 ± 0.67^b^	0.95 ± 0.04^c^	2.74 ± 0.57^b^	12.86 ± 2.57^a^	1.43 ± 0.24^b^	5.44 ± 1.67^ab^
Heptanoic acid, methyl ester	12	1023	170.19 ± 1.67^a^	108.63 ± 28.07^ab^	98.22 ± 41.19^ab^	84.20 ± 22.90^b^	138.94 ± 98.56^a^	93.27 ± 12.89^b^
Butanoic acid, methyl ester	13	724	0.55 ± 0.21	ND	ND	ND	ND	ND
Hexadecanoic acid, methyl ester	14	1915	ND	ND	64.29 ± 8.35^a^	ND	ND	92.20 ± 15.70^a^
Hexanoic acid, 5-methyl-, methyl ester	15	1029	ND	ND	ND	20.80 ± 2.26^a^	11.44 ± 2.90^a^	ND
Oxamic acid, benzyl-, ethyl ester	16	865	ND	6.54 ± 4.88^a^	ND	ND	2.77 ± 1.12^a^	5.05 ± 0.86^a^

Acids								
2-Methylheptanoic acid	17	1058	1.79 ± 0.98	ND	ND	ND	ND	ND
Oxalic acid	18	N.A.	ND	ND	ND	53.42 ± 7.08^a^	21.70 ± 8.02^b^	35.24 ± 24.29^ab^
Lactic acid	19	N.A.	ND	ND	ND	ND	2.95 ± 0.70	ND
Hexanoic acid, 2-ethyl-	20	1167	ND	ND	ND	12.74 ± 0.34	ND	ND
Ketones								
2-Nonanone	21	710	ND	ND	6.11 ± 4.22^a^	ND	ND	11.05 ± 8.48^a^
Acetoin	22	718	ND	ND	ND	53.72 ± 18.50^a^	37.11 ± 10.74^a^	67.13 ± 1.45^a^
5-Hepten-2-one, 6-methyl-	23	986	ND	ND	ND	1.88 ± 0.26	ND	ND

Amines								
Ethanamine, *N*-methyl-	24	N.A.	ND	21.51 ± 7.28^a^	8.90 ± 0.05^a^	ND	ND	ND
sec-Butylamine	25	N.A.	ND	ND	ND	ND	20.17 ± 1.44^a^	4.92 ± 1.58^b^
Dimethylamine	26	426	ND	ND	ND	6.09 ± 1.48	ND	ND

Alcohols								
1-Butanol, 3-methyl-	27	732	ND	ND	ND	9.10 ± 0.05^a^	9.06 ± 3.91^a^	11.82 ± 7.57^a^
1-Octen-4-ol	28	1132	0.92 ± 0.80	ND	ND	ND	ND	ND
Benzeneethanol,.alpha.-methyl-	29	761	ND	4.63 ± 0.32	ND	ND	ND	ND
1-Pentanol	30	760	ND	ND	ND	27.69 ± 5.09^a^	21.94 ± 12.67^a^	11.93 ± 1.27^a^

Note: 1) Different lowercase letters indicate significant differences (p < 0.05); 2) ND indicates not detected; 3) RI: Retention index on DB-5 column or obtained from online databases, https://webbook.nist.gov; 4) N.A.: Not available, indicating no valid retention index data was acquired for the compound.

Aldehydes play a crucial role in the flavor profile of chicken meat due to their low threshold values (Jin et al., 2021). They are primarily produced through the oxidation and degradation of unsaturated fatty acids such as linoleic acid and alpha-linolenic acid (Li et al., 2022). Among aldehydes, hexanal serves as an indicator of lipid oxidation and flavor development, making it a useful measure of chicken meat's oxidative stability and flavor acceptability (Yin et al., 2021). On day 7, the semi-quantitative equivalents of hexanal and nonanal in the WT group were significantly higher than in the Δ*Hfq* group (*p* < 0.05), contributing to a grassy and fatty taste in the chicken. This suggests that WT strains accelerate the oxidative process in chicken. Alkanes are primarily formed through the degradation of fatty acids and lipid oxidation (Fu et al., 2022). Previous studies have reported a negative correlation between the relative concentrations of aldehydes and alkanes in meat (Jin et al., 2021), which is consistent with the findings of this study. Esters are produced during microbial metabolism, where fatty acids and carboxylic acids react with alcohols (Lei et al., 2022a). Among all esters, the WT group showed significantly higher semi-quantitative equivalents of heptanoic acid, methyl ester compared to the control and Δ*Hfq* groups. This is likely associated with lipase secretion by WT strains, which may contribute to enhanced fat hydrolysis, releasing more free fatty acids such as heptanoic acid for esterification. Acids may be produced through microbial degradation of sugars and amino acids. In the later stages of spoilage, all three groups produced oxalic acid, with the WT group showing significantly higher levels than the control and Δ*Hfq* groups. However, because acids have higher threshold values, they contribute less to the overall odor. Ketones, typically formed through the oxidation of amino acids and lipids, were also observed. Among the ketones detected, acetoin was not present in the early stages but was found in all three groups by day 7. Acetoin has been identified as a spoilage marker in chicken meat (Casaburi et al., 2014). Amines are primarily produced through microbial decarboxylase activity on free amino acids, leading to nitrogenous bases production (Jairath et al., 2015). Sec-butylamine was only detected in the inoculated groups at day 7. The WT group produced 20.17 μg/kg (semi-quantitative equivalent) of sec-butylamine, significantly higher than the 4.92 μg/kg (semi-quantitative equivalent) found in the Δ*Hfq* group. This suggests that sec-butylamine is not an endogenous product of chicken meat but a metabolite resulting from contamination by *P. agglomerans*. The lower semi-quantitative equivalents of sec-butylamine in the Δ*Hfq* group may be associated with altered expression of amino acid decarboxylase genes, potentially correlating with its synthesis under the present model. Alcohols, typically produced through the degradation of linoleic acid in muscle tissues by lipoxygenase and peroxidase, were also detected in later stages of storage. 1-butanol, 3-methyl- and 1-pentanol were identified, with 1-butanol, 3-methyl- being a characteristic compound of microbial contamination in beef (Hernández-Macedo et al., 2012). To verify the reliability of GC–MS qualitative results, key markers such as acetoin and sec-butylamine were verified using authentic standards. Mass spectral analysis showed that the characteristic fragment ions of acetoin and sec-butylamine in samples were highly consistent with those of the standard spectra. Specifically, diagnostic fragment ions at *m*/*z* 45.0 and 88.0 for acetoin, and at m/z 44.0 and 58.0 for sec-butylamine, matched well between samples and standards, confirming the accuracy of the identification. All standard solutions and sample extracts were analyzed in separate analytical batches. Random retention-time fluctuation derived from inter-batch instrumental drift precluded retention time from being adopted for qualitative confirmation, and characteristic fragment-ion matching was defined as the exclusive identification criterion in this study (Fig. S2 and S3).

### 3.10 Partial correlation analysis

Partial correlation analysis was performed on pH, TVB-N, TBA, texture, *E*-nose reading, E-tongue responses, and GC–MS indexes using the Pearson correlation method in SPSS, with storage time controlled as the covariate. The correlation matrix was established based on *N* = 15 independent samples. All correlation *p*-values were adjusted for multiple comparisons. The results presented in Table 4 indicate that pH has a strong positive correlation with TVB-N, and TVB-N is similarly strongly correlated with TBA. Furthermore, the E-nose sensors W1W, W6S and W5S exhibited strong positive correlations with one another, as well as with pH, TVB-N and TBA. This suggests that variations in the E-nose responses are closely related to the quality deterioration of chicken during cold storage under the present UV-treated in situ model. Regarding texture, only hardness demonstrated a strong positive correlation with chewiness, highlighting hardness as the primary textural factor influencing chewiness. Among the flavor indicators, umami showed a strong positive correlation with bitterness. Additionally, heptane was positively correlated with TVB-N, TBA and sensor W6S, while exhibiting a negative correlation with umami and bitterness. These observations imply that alterations in flavor compounds are associated with chicken quality deterioration as well as E-nose responses under the present experimental system.

**Table 4:** Partial correlation analysis of various indicators in chicken meat stored at 4°C.

Indicators	pH	TVB-N	TBA	Hardness	Springiness	Chewiness	Cohesiveness	W1W	W6S	W5S	Umami	Bitterness	Heptane
PH	1.000												
TVB-N	0.915^⁎⁎⁎^	1.000											
TBA	0.887	0.974^⁎⁎⁎^	1.000										
Hardness	−0.668	−0.673	−0.785	1.000									
Springiness	−0.509	−0.685	−0.674	0.587	1.000								
Chewiness	−0.721	−0.811	−0.89	0.866^⁎⁎^	0.664	1.000							
Cohesiveness	−0.662	−0.807	−0.865^⁎⁎^	0.575	0.406	0.708	1.000						
W1W	0.946^⁎⁎⁎^	0.924^⁎⁎⁎^	0.945^⁎⁎⁎^	−0.795	−0.607	−0.849	−0.741	1.000					
W6S	0.876^⁎⁎^	0.794^⁎^	0.777^⁎^	−0.657	−0.482	−0.586	−0.511	0.894^⁎⁎⁎^	1.000				
W5S	0.953^⁎⁎⁎^	0.941^⁎⁎⁎^	0.956^⁎⁎⁎^	−0.792	−0.621	−0.85	−0.751	0.999^⁎⁎⁎^	0.893^⁎⁎⁎^	1.000			
Umami	−0.768	−0.817^⁎⁎^	−0.757^⁎^	0.478	0.497	0.426	0.601	−0.726	−0.85	−0.749	1.000		
Bitterness	−0.883	−0.92^⁎⁎⁎^	−0.844	0.487	0.486	0.589	0.656	−0.803	−0.809	−0.828	0.913^⁎⁎⁎^	1.000	
Heptane	0.78	0.899^⁎⁎⁎^	0.832^⁎⁎^	−0.571	−0.693	−0.604	−0.611	0.739	0.74^⁎^	0.771	−0.915^⁎⁎⁎^	−0.929^⁎⁎⁎^	1.000

Note: *p < 0.05, **p < 0.01, ***p < 0.001.

## 4 Conclusion

This study suggests that *P. agglomerans* may contribute to the accelerated spoilage of chilled chicken under the present UV-treated in situ model, as indicated by changes in microbial indicators, pH, TVB-N, TBA, texture, and the accumulation of volatile compounds, such as aldehydes and sec-butylamine. These changes may also alter the odor and taste of the chicken. In contrast, the absence of the *Hfq* gene may be associated with reduced spoilage effects, suggesting that *P. agglomerans* may play a key role in spoilage and that Hfq may regulate this process. However, since a complemented strain (Δ*Hfq*::*hfq*) was not included in this study, the genetic causality underlying these observations remains to be further confirmed. These findings provide valuable insights into the spoilage mechanisms of chicken under the present UV-treated in situ model and form a basis for developing preservation technologies.

## CRediT authorship contribution statement

**Xiaochen Wang:** Writing – original draft, Validation, Methodology, Investigation, Data curation, Conceptualization. **Yuhang Ma:** Methodology. **Yafeng Wang:** Data curation. **Lianxin Qi:** Data curation. **Yangyong Lv:** Writing – review & editing. **Shan Wei:** Writing – review & editing. **Fengguang Zhao:** Resources. **Yingying Wang:** Resources. **Shuaibing Zhang:** Writing – review & editing. **Yang Lei:** Supervision, Project administration, Funding acquisition. **Yuansen Hu:** Supervision, Project administration, Funding acquisition. **Ming Huang:** Funding acquisition.

## Declaration of competing interest

The authors declare that they have no known competing financial interests or personal relationships that could have appeared to influence the work reported in this paper.

## Data Availability

Data will be made available on request.
