# Supplementary material for: Study on the impact of Hfq regulation on spoilage odor formation by Pantoea agglomerans inoculated onto chilled chicken meat
Source: Food Chem X. 2026 May 30;37:104052. doi: 10.1016/j.fochx.2026.104052 (PMC13265691; doi:10.1016/j.fochx.2026.104052)
Supplement: Supplementary material [file mmc1.docx]

**Table S1** Homology analysis of 16S rRNA sequences of inoculated strains during chilled chicken storage.

| Sample ID | BLAST identification | Sequence length (bp) | Query coverage (%) | Per. ident  (%) | Accession |
| --- | --- | --- | --- | --- | --- |
| WT-0d-1 | *Pantoea agglomerans* | 1380 | 100 | 99.06 | NR_041978.1 |
| WT-0d-2 | *Pantoea agglomerans* | 1370 | 100 | 99.05 | NR_041978.1 |
| WT-0d-3 | *Pantoea agglomerans* | 1383 | 100 | 99.06 | NR_041978.1 |
| Δ*Hfq*-0d-1 | *Pantoea agglomerans* | 1375 | 100 | 99.05 | NR_041978.1 |
| Δ*Hfq*-0d-2 | *Pantoea agglomerans* | 1381 | 100 | 99.05 | NR_041978.1 |
| Δ*Hfq*-0d-3 | *Pantoea agglomerans* | 1365 | 100 | 99.06 | NR_041978.1 |
| WT-1d-1 | *Pantoea agglomerans* | 1366 | 100 | 99.05 | NR_041978.1 |
| WT-1d-2 | *Pantoea agglomerans* | 1374 | 100 | 99.05 | NR_041978.1 |
| WT-1d-3 | *Pantoea agglomerans* | 1376 | 100 | 99.06 | NR_041978.1 |
| Δ*Hfq*-1d-1 | *Pantoea agglomerans* | 1380 | 100 | 99.06 | NR_041978.1 |
| Δ*Hfq*-1d-2 | *Pantoea agglomerans* | 1387 | 100 | 99.06 | NR_041978.1 |
| Δ*Hfq*-1d-3 | *Pantoea agglomerans* | 1383 | 100 | 99.06 | NR_041978.1 |
| WT-3d-1 | *Pantoea agglomerans* | 1366 | 100 | 99.05 | NR_041978.1 |
| WT-3d-2 | *Pantoea agglomerans* | 1364 | 100 | 99.05 | NR_041978.1 |
| WT-3d-3 | *Pantoea agglomerans* | 1368 | 100 | 99.05 | NR_041978.1 |
| Δ*Hfq*-3d-1 | *Pantoea agglomerans* | 1364 | 100 | 99.05 | NR_041978.1 |
| Δ*Hfq*-3d-2 | *Pantoea agglomerans* | 1366 | 100 | 99.05 | NR_041978.1 |
| Δ*Hfq*-3d-3 | *Pantoea agglomerans* | 1366 | 100 | 99.05 | NR_041978.1 |
| WT-5d-1 | *Pantoea agglomerans* | 1378 | 100 | 99.06 | NR_041978.1 |
| WT-5d-2 | *Pantoea agglomerans* | 1374 | 100 | 99.05 | NR_041978.1 |
| WT-5d-3 | *Pantoea agglomerans* | 1367 | 100 | 99.05 | NR_041978.1 |
| Δ*Hfq*-5d-1 | *Pantoea agglomerans* | 1366 | 100 | 99.05 | NR_041978.1 |
| Δ*Hfq*-5d-2 | *Pantoea agglomerans* | 1367 | 100 | 99.05 | NR_041978.1 |
| Δ*Hfq*-5d-3 | *Pantoea agglomerans* | 1365 | 100 | 99.05 | NR_041978.1 |
| WT-7d-1 | *Pantoea agglomerans* | 1365 | 100 | 99.05 | NR_041978.1 |
| WT-7d-2 | *Pantoea agglomerans* | 1365 | 100 | 99.05 | NR_041978.1 |
| WT-7d-3 | *Pantoea agglomerans* | 1366 | 100 | 99.05 | NR_041978.1 |
| Δ*Hfq*-7d-1 | *Pantoea agglomerans* | 1367 | 100 | 99.05 | NR_041978.1 |
| Δ*Hfq*-7d-2 | *Pantoea agglomerans* | 1365 | 100 | 99.05 | NR_041978.1 |
| Δ*Hfq*-7d-3 | *Pantoea agglomerans* | 1368 | 100 | 99.05 | NR_041978.1 |

Note: The E-values for all samples are 0.0.

**Raw 16S rRNA gene sequences of single colony isolates from WT and Δ*Hfq* groups sampled on storage days 0, 1, 3, 5 and 7.**

**WT-0d-1:**

CACAGAGGAGCTTGCTCCTTGGGTGACGAGTGGCGGACGGGTGAGTAATGTCTGGGGATCTGCCCGATAGAGGGGGATAACCACTGGAAACGGTGGCTAATACCGCATAACGTCGCAAGACCAAAGAGGGGGACCTTCGGGCCTCTCACTATCGGATGAACCCAGATGGGATTAGCTAGTAGGCGGGGTAATGGCCCACCTAGGCGACGATCCCTAGCTGGTCTGAGAGGATGACCAGCCACACTGGAACTGAGACACGGTCCAGACTCCTACGGGAGGCAGCAGTGGGGAATATTGCACAATGGGCGCAAGCCTGATGCAGCCATGCCGCGTGTATGAAGAAGGCCTTCGGGTTGTAAAGTACTTTCAGCGGGGAGGAAGGCGATGTGGTTAATAACCGCGTCGATTGACGTTACCCGCAGAAGAAGCACCGGCTAACTCCGTGCCAGCAGCCGCGGTAATACGGAGGGTGCAAGCGTTAATCGGAATTACTGGGCGTAAAGCGCACGCAGGCGGTCTGTTAAGTCAGATGTGAAATCCCCGGGCTTAACCTGGGAACTGCATTTGAAACTGGCAGGCTTGAGTCTTGTAGAGGGGGGTAGAATTCCAGGTGTAGCGGTGAAATGCGTAGAGATCTGGAGGAATACCGGTGGCGAAGGCGGCCCCCTGGACAAAGACTGACGCTCAGGTGCGAAAGCGTGGGGAGCAAACAGGATTAGATACCCTGGTAGTCCACGCCGTAAACGATGTCGACTTGGAGGTTGTTCCCTTGAGGAGTGGCTTCCGGAGCTAACGCGTTAAGTCGACCGCCTGGGGAGTACGGCCGCAAGGTTAAAACTCAAATGAATTGACGGGGGCCCGCACAAGCGGTGGAGCATGTGGTTTAATTCGATGCAACGCGAAGAACCTTACCTACTCTTGACATCCAGCGAACTTTCCAGAGATGGATTGGTGCCTTCGGGAACGCTGAGACAGGTGCTGCATGGCTGTCGTCAGCTCGTGTTGTGAAATGTTGGGTTAAGTCCCGCAACGAGCGCAACCCTTATCCTTTGTTGCCAGCGATTCGGTCGGGAACTCAAAGGAGACTGCCGGTGATAAACCGGAGGAAGGTGGGGATGACGTCAAGTCATCATGGCCCTTACGAGTAGGGCTACACACGTGCTACAATGGCGCATACAAAGAGAAGCGACCTCGCGAGAGCAAGCGGACCTCACAAAGTGCGTCGTAGTCCGGATCGGAGTCTGCAACTCGACTCCGTGAAGTCGGAATCGCTAGTAATCGTGGATCAGAATGCCACGGTGAATACGTTCCCGGGCCTTGTACACACCGCCCGTCACACCATGGGAGTGGGTTGCAAAAGAAGTAGGTAGCTTAACCTTC

**WT-0d-2:**

GGAGCTTGCTCCTTGGGTGACGAGTGGCGGACGGGTGAGTAATGTCTGGGGATCTGCCCGATAGAGGGGGATAACCACTGGAAACGGTGGCTAATACCGCATAACGTCGCAAGACCAAAGAGGGGGACCTTCGGGCCTCTCACTATCGGATGAACCCAGATGGGATTAGCTAGTAGGCGGGGTAATGGCCCACCTAGGCGACGATCCCTAGCTGGTCTGAGAGGATGACCAGCCACACTGGAACTGAGACACGGTCCAGACTCCTACGGGAGGCAGCAGTGGGGAATATTGCACAATGGGCGCAAGCCTGATGCAGCCATGCCGCGTGTATGAAGAAGGCCTTCGGGTTGTAAAGTACTTTCAGCGGGGAGGAAGGCGATGTGGTTAATAACCGCGTCGATTGACGTTACCCGCAGAAGAAGCACCGGCTAACTCCGTGCCAGCAGCCGCGGTAATACGGAGGGTGCAAGCGTTAATCGGAATTACTGGGCGTAAAGCGCACGCAGGCGGTCTGTTAAGTCAGATGTGAAATCCCCGGGCTTAACCTGGGAACTGCATTTGAAACTGGCAGGCTTGAGTCTTGTAGAGGGGGGTAGAATTCCAGGTGTAGCGGTGAAATGCGTAGAGATCTGGAGGAATACCGGTGGCGAAGGCGGCCCCCTGGACAAAGACTGACGCTCAGGTGCGAAAGCGTGGGGAGCAAACAGGATTAGATACCCTGGTAGTCCACGCCGTAAACGATGTCGACTTGGAGGTTGTTCCCTTGAGGAGTGGCTTCCGGAGCTAACGCGTTAAGTCGACCGCCTGGGGAGTACGGCCGCAAGGTTAAAACTCAAATGAATTGACGGGGGCCCGCACAAGCGGTGGAGCATGTGGTTTAATTCGATGCAACGCGAAGAACCTTACCTACTCTTGACATCCAGCGAACTTTCCAGAGATGGATTGGTGCCTTCGGGAACGCTGAGACAGGTGCTGCATGGCTGTCGTCAGCTCGTGTTGTGAAATGTTGGGTTAAGTCCCGCAACGAGCGCAACCCTTATCCTTTGTTGCCAGCGATTCGGTCGGGAACTCAAAGGAGACTGCCGGTGATAAACCGGAGGAAGGTGGGGATGACGTCAAGTCATCATGGCCCTTACGAGTAGGGCTACACACGTGCTACAATGGCGCATACAAAGAGAAGCGACCTCGCGAGAGCAAGCGGACCTCACAAAGTGCGTCGTAGTCCGGATCGGAGTCTGCAACTCGACTCCGTGAAGTCGGAATCGCTAGTAATCGTGGATCAGAATGCCACGGTGAATACGTTCCCGGGCCTTGTACACACCGCCCGTCACACCATGGGAGTGGGTTGCAAAAGAAGTAGGTAGCTTAAC

**WT-0d-3:**

CACAGAGGAGCTTGCTCCTTGGGTGACGAGTGGCGGACGGGTGAGTAATGTCTGGGGATCTGCCCGATAGAGGGGGATAACCACTGGAAACGGTGGCTAATACCGCATAACGTCGCAAGACCAAAGAGGGGGACCTTCGGGCCTCTCACTATCGGATGAACCCAGATGGGATTAGCTAGTAGGCGGGGTAATGGCCCACCTAGGCGACGATCCCTAGCTGGTCTGAGAGGATGACCAGCCACACTGGAACTGAGACACGGTCCAGACTCCTACGGGAGGCAGCAGTGGGGAATATTGCACAATGGGCGCAAGCCTGATGCAGCCATGCCGCGTGTATGAAGAAGGCCTTCGGGTTGTAAAGTACTTTCAGCGGGGAGGAAGGCGATGTGGTTAATAACCGCGTCGATTGACGTTACCCGCAGAAGAAGCACCGGCTAACTCCGTGCCAGCAGCCGCGGTAATACGGAGGGTGCAAGCGTTAATCGGAATTACTGGGCGTAAAGCGCACGCAGGCGGTCTGTTAAGTCAGATGTGAAATCCCCGGGCTTAACCTGGGAACTGCATTTGAAACTGGCAGGCTTGAGTCTTGTAGAGGGGGGTAGAATTCCAGGTGTAGCGGTGAAATGCGTAGAGATCTGGAGGAATACCGGTGGCGAAGGCGGCCCCCTGGACAAAGACTGACGCTCAGGTGCGAAAGCGTGGGGAGCAAACAGGATTAGATACCCTGGTAGTCCACGCCGTAAACGATGTCGACTTGGAGGTTGTTCCCTTGAGGAGTGGCTTCCGGAGCTAACGCGTTAAGTCGACCGCCTGGGGAGTACGGCCGCAAGGTTAAAACTCAAATGAATTGACGGGGGCCCGCACAAGCGGTGGAGCATGTGGTTTAATTCGATGCAACGCGAAGAACCTTACCTACTCTTGACATCCAGCGAACTTTCCAGAGATGGATTGGTGCCTTCGGGAACGCTGAGACAGGTGCTGCATGGCTGTCGTCAGCTCGTGTTGTGAAATGTTGGGTTAAGTCCCGCAACGAGCGCAACCCTTATCCTTTGTTGCCAGCGATTCGGTCGGGAACTCAAAGGAGACTGCCGGTGATAAACCGGAGGAAGGTGGGGATGACGTCAAGTCATCATGGCCCTTACGAGTAGGGCTACACACGTGCTACAATGGCGCATACAAAGAGAAGCGACCTCGCGAGAGCAAGCGGACCTCACAAAGTGCGTCGTAGTCCGGATCGGAGTCTGCAACTCGACTCCGTGAAGTCGGAATCGCTAGTAATCGTGGATCAGAATGCCACGGTGAATACGTTCCCGGGCCTTGTACACACCGCCCGTCACACCATGGGAGTGGGTTGCAAAAGAAGTAGGTAGCTTAACCTTCGGG

**Δ*Hfq*-0d-1:**

CACAGAGGAGCTTGCTCCTTGGGTGACGAGTGGCGGACGGGTGAGTAATGTCTGGGGATCTGCCCGATAGAGGGGGATAACCACTGGAAACGGTGGCTAATACCGCATAACGTCGCAAGACCAAAGAGGGGGACCTTCGGGCCTCTCACTATCGGATGAACCCAGATGGGATTAGCTAGTAGGCGGGGTAATGGCCCACCTAGGCGACGATCCCTAGCTGGTCTGAGAGGATGACCAGCCACACTGGAACTGAGACACGGTCCAGACTCCTACGGGAGGCAGCAGTGGGGAATATTGCACAATGGGCGCAAGCCTGATGCAGCCATGCCGCGTGTATGAAGAAGGCCTTCGGGTTGTAAAGTACTTTCAGCGGGGAGGAAGGCGATGTGGTTAATAACCGCGTCGATTGACGTTACCCGCAGAAGAAGCACCGGCTAACTCCGTGCCAGCAGCCGCGGTAATACGGAGGGTGCAAGCGTTAATCGGAATTACTGGGCGTAAAGCGCACGCAGGCGGTCTGTTAAGTCAGATGTGAAATCCCCGGGCTTAACCTGGGAACTGCATTTGAAACTGGCAGGCTTGAGTCTTGTAGAGGGGGGTAGAATTCCAGGTGTAGCGGTGAAATGCGTAGAGATCTGGAGGAATACCGGTGGCGAAGGCGGCCCCCTGGACAAAGACTGACGCTCAGGTGCGAAAGCGTGGGGAGCAAACAGGATTAGATACCCTGGTAGTCCACGCCGTAAACGATGTCGACTTGGAGGTTGTTCCCTTGAGGAGTGGCTTCCGGAGCTAACGCGTTAAGTCGACCGCCTGGGGAGTACGGCCGCAAGGTTAAAACTCAAATGAATTGACGGGGGCCCGCACAAGCGGTGGAGCATGTGGTTTAATTCGATGCAACGCGAAGAACCTTACCTACTCTTGACATCCAGCGAACTTTCCAGAGATGGATTGGTGCCTTCGGGAACGCTGAGACAGGTGCTGCATGGCTGTCGTCAGCTCGTGTTGTGAAATGTTGGGTTAAGTCCCGCAACGAGCGCAACCCTTATCCTTTGTTGCCAGCGATTCGGTCGGGAACTCAAAGGAGACTGCCGGTGATAAACCGGAGGAAGGTGGGGATGACGTCAAGTCATCATGGCCCTTACGAGTAGGGCTACACACGTGCTACAATGGCGCATACAAAGAGAAGCGACCTCGCGAGAGCAAGCGGACCTCACAAAGTGCGTCGTAGTCCGGATCGGAGTCTGCAACTCGACTCCGTGAAGTCGGAATCGCTAGTAATCGTGGATCAGAATGCCACGGTGAATACGTTCCCGGGCCTTGTACACACCGCCCGTCACACCATGGGAGTGGGTTGCAAAAGAAGTAGGTAGCTTAA

**Δ*Hfq*-0d-2:**

AGCACAGAGGAGCTTGCTCCTTGGGTGACGAGTGGCGGACGGGTGAGTAATGTCTGGGGATCTGCCCGATAGAGGGGGATAACCACTGGAAACGGTGGCTAATACCGCATAACGTCGCAAGACCAAAGAGGGGGACCTTCGGGCCTCTCACTATCGGATGAACCCAGATGGGATTAGCTAGTAGGCGGGGTAATGGCCCACCTAGGCGACGATCCCTAGCTGGTCTGAGAGGATGACCAGCCACACTGGAACTGAGACACGGTCCAGACTCCTACGGGAGGCAGCAGTGGGGAATATTGCACAATGGGCGCAAGCCTGATGCAGCCATGCCGCGTGTATGAAGAAGGCCTTCGGGTTGTAAAGTACTTTCAGCGGGGAGGAAGGCGATGTGGTTAATAACCGCGTCGATTGACGTTACCCGCAGAAGAAGCACCGGCTAACTCCGTGCCAGCAGCCGCGGTAATACGGAGGGTGCAAGCGTTAATCGGAATTACTGGGCGTAAAGCGCACGCAGGCGGTCTGTTAAGTCAGATGTGAAATCCCCGGGCTTAACCTGGGAACTGCATTTGAAACTGGCAGGCTTGAGTCTTGTAGAGGGGGGTAGAATTCCAGGTGTAGCGGTGAAATGCGTAGAGATCTGGAGGAATACCGGTGGCGAAGGCGGCCCCCTGGACAAAGACTGACGCTCAGGTGCGAAAGCGTGGGGAGCAAACAGGATTAGATACCCTGGTAGTCCACGCCGTAAACGATGTCGACTTGGAGGTTGTTCCCTTGAGGAGTGGCTTCCGGAGCTAACGCGTTAAGTCGACCGCCTGGGGAGTACGGCCGCAAGGTTAAAACTCAAATGAATTGACGGGGGCCCGCACAAGCGGTGGAGCATGTGGTTTAATTCGATGCAACGCGAAGAACCTTACCTACTCTTGACATCCAGCGAACTTTCCAGAGATGGATTGGTGCCTTCGGGAACGCTGAGACAGGTGCTGCATGGCTGTCGTCAGCTCGTGTTGTGAAATGTTGGGTTAAGTCCCGCAACGAGCGCAACCCTTATCCTTTGTTGCCAGCGATTCGGTCGGGAACTCAAAGGAGACTGCCGGTGATAAACCGGAGGAAGGTGGGGATGACGTCAAGTCATCATGGCCCTTACGAGTAGGGCTACACACGTGCTACAATGGCGCATACAAAGAGAAGCGACCTCGCGAGAGCAAGCGGACCTCACAAAGTGCGTCGTAGTCCGGATCGGAGTCTGCAACTCGACTCCGTGAAGTCGGAATCGCTAGTAATCGTGGATCAGAATGCCACGGTGAATACGTTCCCGGGCCTTGTACACACCGCCCGTCACACCATGGGAGTGGGTTGCAAAAGAAGTAGGTAGCTTAACCTT

**Δ*Hfq*-0d-3:**

ACAGAGGAGCTTGCTCCTTGGGTGACGAGTGGCGGACGGGTGAGTAATGTCTGGGGATCTGCCCGATAGAGGGGGATAACCACTGGAAACGGTGGCTAATACCGCATAACGTCGCAAGACCAAAGAGGGGGACCTTCGGGCCTCTCACTATCGGATGAACCCAGATGGGATTAGCTAGTAGGCGGGGTAATGGCCCACCTAGGCGACGATCCCTAGCTGGTCTGAGAGGATGACCAGCCACACTGGAACTGAGACACGGTCCAGACTCCTACGGGAGGCAGCAGTGGGGAATATTGCACAATGGGCGCAAGCCTGATGCAGCCATGCCGCGTGTATGAAGAAGGCCTTCGGGTTGTAAAGTACTTTCAGCGGGGAGGAAGGCGATGTGGTTAATAACCGCGTCGATTGACGTTACCCGCAGAAGAAGCACCGGCTAACTCCGTGCCAGCAGCCGCGGTAATACGGAGGGTGCAAGCGTTAATCGGAATTACTGGGCGTAAAGCGCACGCAGGCGGTCTGTTAAGTCAGATGTGAAATCCCCGGGCTTAACCTGGGAACTGCATTTGAAACTGGCAGGCTTGAGTCTTGTAGAGGGGGGTAGAATTCCAGGTGTAGCGGTGAAATGCGTAGAGATCTGGAGGAATACCGGTGGCGAAGGCGGCCCCCTGGACAAAGACTGACGCTCAGGTGCGAAAGCGTGGGGAGCAAACAGGATTAGATACCCTGGTAGTCCACGCCGTAAACGATGTCGACTTGGAGGTTGTTCCCTTGAGGAGTGGCTTCCGGAGCTAACGCGTTAAGTCGACCGCCTGGGGAGTACGGCCGCAAGGTTAAAACTCAAATGAATTGACGGGGGCCCGCACAAGCGGTGGAGCATGTGGTTTAATTCGATGCAACGCGAAGAACCTTACCTACTCTTGACATCCAGCGAACTTTCCAGAGATGGATTGGTGCCTTCGGGAACGCTGAGACAGGTGCTGCATGGCTGTCGTCAGCTCGTGTTGTGAAATGTTGGGTTAAGTCCCGCAACGAGCGCAACCCTTATCCTTTGTTGCCAGCGATTCGGTCGGGAACTCAAAGGAGACTGCCGGTGATAAACCGGAGGAAGGTGGGGATGACGTCAAGTCATCATGGCCCTTACGAGTAGGGCTACACACGTGCTACAATGGCGCATACAAAGAGAAGCGACCTCGCGAGAGCAAGCGGACCTCACAAAGTGCGTCGTAGTCCGGATCGGAGTCTGCAACTCGACTCCGTGAAGTCGGAATCGCTAGTAATCGTGGATCAGAATGCCACGGTGAATACGTTCCCGGGCCTTGTACACACCGCCCGTCACACCATGGGAGTGGGTTGCAAAAGAAGTAG

**WT-1d-1:**

CACAGAGGAGCTTGCTCCTTGGGTGACGAGTGGCGGACGGGTGAGTAATGTCTGGGGATCTGCCCGATAGAGGGGGATAACCACTGGAAACGGTGGCTAATACCGCATAACGTCGCAAGACCAAAGAGGGGGACCTTCGGGCCTCTCACTATCGGATGAACCCAGATGGGATTAGCTAGTAGGCGGGGTAATGGCCCACCTAGGCGACGATCCCTAGCTGGTCTGAGAGGATGACCAGCCACACTGGAACTGAGACACGGTCCAGACTCCTACGGGAGGCAGCAGTGGGGAATATTGCACAATGGGCGCAAGCCTGATGCAGCCATGCCGCGTGTATGAAGAAGGCCTTCGGGTTGTAAAGTACTTTCAGCGGGGAGGAAGGCGATGTGGTTAATAACCGCGTCGATTGACGTTACCCGCAGAAGAAGCACCGGCTAACTCCGTGCCAGCAGCCGCGGTAATACGGAGGGTGCAAGCGTTAATCGGAATTACTGGGCGTAAAGCGCACGCAGGCGGTCTGTTAAGTCAGATGTGAAATCCCCGGGCTTAACCTGGGAACTGCATTTGAAACTGGCAGGCTTGAGTCTTGTAGAGGGGGGTAGAATTCCAGGTGTAGCGGTGAAATGCGTAGAGATCTGGAGGAATACCGGTGGCGAAGGCGGCCCCCTGGACAAAGACTGACGCTCAGGTGCGAAAGCGTGGGGAGCAAACAGGATTAGATACCCTGGTAGTCCACGCCGTAAACGATGTCGACTTGGAGGTTGTTCCCTTGAGGAGTGGCTTCCGGAGCTAACGCGTTAAGTCGACCGCCTGGGGAGTACGGCCGCAAGGTTAAAACTCAAATGAATTGACGGGGGCCCGCACAAGCGGTGGAGCATGTGGTTTAATTCGATGCAACGCGAAGAACCTTACCTACTCTTGACATCCAGCGAACTTTCCAGAGATGGATTGGTGCCTTCGGGAACGCTGAGACAGGTGCTGCATGGCTGTCGTCAGCTCGTGTTGTGAAATGTTGGGTTAAGTCCCGCAACGAGCGCAACCCTTATCCTTTGTTGCCAGCGATTCGGTCGGGAACTCAAAGGAGACTGCCGGTGATAAACCGGAGGAAGGTGGGGATGACGTCAAGTCATCATGGCCCTTACGAGTAGGGCTACACACGTGCTACAATGGCGCATACAAAGAGAAGCGACCTCGCGAGAGCAAGCGGACCTCACAAAGTGCGTCGTAGTCCGGATCGGAGTCTGCAACTCGACTCCGTGAAGTCGGAATCGCTAGTAATCGTGGATCAGAATGCCACGGTGAATACGTTCCCGGGCCTTGTACACACCGCCCGTCACACCATGGGAGTGGGTTGCAAAAGAAGTAG

**WT-1d-2:**

TAGCACAGAGGAGCTTGCTCCTTGGGTGACGAGTGGCGGACGGGTGAGTAATGTCTGGGGATCTGCCCGATAGAGGGGGATAACCACTGGAAACGGTGGCTAATACCGCATAACGTCGCAAGACCAAAGAGGGGGACCTTCGGGCCTCTCACTATCGGATGAACCCAGATGGGATTAGCTAGTAGGCGGGGTAATGGCCCACCTAGGCGACGATCCCTAGCTGGTCTGAGAGGATGACCAGCCACACTGGAACTGAGACACGGTCCAGACTCCTACGGGAGGCAGCAGTGGGGAATATTGCACAATGGGCGCAAGCCTGATGCAGCCATGCCGCGTGTATGAAGAAGGCCTTCGGGTTGTAAAGTACTTTCAGCGGGGAGGAAGGCGATGTGGTTAATAACCGCGTCGATTGACGTTACCCGCAGAAGAAGCACCGGCTAACTCCGTGCCAGCAGCCGCGGTAATACGGAGGGTGCAAGCGTTAATCGGAATTACTGGGCGTAAAGCGCACGCAGGCGGTCTGTTAAGTCAGATGTGAAATCCCCGGGCTTAACCTGGGAACTGCATTTGAAACTGGCAGGCTTGAGTCTTGTAGAGGGGGGTAGAATTCCAGGTGTAGCGGTGAAATGCGTAGAGATCTGGAGGAATACCGGTGGCGAAGGCGGCCCCCTGGACAAAGACTGACGCTCAGGTGCGAAAGCGTGGGGAGCAAACAGGATTAGATACCCTGGTAGTCCACGCCGTAAACGATGTCGACTTGGAGGTTGTTCCCTTGAGGAGTGGCTTCCGGAGCTAACGCGTTAAGTCGACCGCCTGGGGAGTACGGCCGCAAGGTTAAAACTCAAATGAATTGACGGGGGCCCGCACAAGCGGTGGAGCATGTGGTTTAATTCGATGCAACGCGAAGAACCTTACCTACTCTTGACATCCAGCGAACTTTCCAGAGATGGATTGGTGCCTTCGGGAACGCTGAGACAGGTGCTGCATGGCTGTCGTCAGCTCGTGTTGTGAAATGTTGGGTTAAGTCCCGCAACGAGCGCAACCCTTATCCTTTGTTGCCAGCGATTCGGTCGGGAACTCAAAGGAGACTGCCGGTGATAAACCGGAGGAAGGTGGGGATGACGTCAAGTCATCATGGCCCTTACGAGTAGGGCTACACACGTGCTACAATGGCGCATACAAAGAGAAGCGACCTCGCGAGAGCAAGCGGACCTCACAAAGTGCGTCGTAGTCCGGATCGGAGTCTGCAACTCGACTCCGTGAAGTCGGAATCGCTAGTAATCGTGGATCAGAATGCCACGGTGAATACGTTCCCGGGCCTTGTACACACCGCCCGTCACACCATGGGAGTGGGTTGCAAAAGAAGTAGGTAGC

**WT-1d-3:**

CACAGAGGAGCTTGCTCCTTGGGTGACGAGTGGCGGACGGGTGAGTAATGTCTGGGGATCTGCCCGATAGAGGGGGATAACCACTGGAAACGGTGGCTAATACCGCATAACGTCGCAAGACCAAAGAGGGGGACCTTCGGGCCTCTCACTATCGGATGAACCCAGATGGGATTAGCTAGTAGGCGGGGTAATGGCCCACCTAGGCGACGATCCCTAGCTGGTCTGAGAGGATGACCAGCCACACTGGAACTGAGACACGGTCCAGACTCCTACGGGAGGCAGCAGTGGGGAATATTGCACAATGGGCGCAAGCCTGATGCAGCCATGCCGCGTGTATGAAGAAGGCCTTCGGGTTGTAAAGTACTTTCAGCGGGGAGGAAGGCGATGTGGTTAATAACCGCGTCGATTGACGTTACCCGCAGAAGAAGCACCGGCTAACTCCGTGCCAGCAGCCGCGGTAATACGGAGGGTGCAAGCGTTAATCGGAATTACTGGGCGTAAAGCGCACGCAGGCGGTCTGTTAAGTCAGATGTGAAATCCCCGGGCTTAACCTGGGAACTGCATTTGAAACTGGCAGGCTTGAGTCTTGTAGAGGGGGGTAGAATTCCAGGTGTAGCGGTGAAATGCGTAGAGATCTGGAGGAATACCGGTGGCGAAGGCGGCCCCCTGGACAAAGACTGACGCTCAGGTGCGAAAGCGTGGGGAGCAAACAGGATTAGATACCCTGGTAGTCCACGCCGTAAACGATGTCGACTTGGAGGTTGTTCCCTTGAGGAGTGGCTTCCGGAGCTAACGCGTTAAGTCGACCGCCTGGGGAGTACGGCCGCAAGGTTAAAACTCAAATGAATTGACGGGGGCCCGCACAAGCGGTGGAGCATGTGGTTTAATTCGATGCAACGCGAAGAACCTTACCTACTCTTGACATCCAGCGAACTTTCCAGAGATGGATTGGTGCCTTCGGGAACGCTGAGACAGGTGCTGCATGGCTGTCGTCAGCTCGTGTTGTGAAATGTTGGGTTAAGTCCCGCAACGAGCGCAACCCTTATCCTTTGTTGCCAGCGATTCGGTCGGGAACTCAAAGGAGACTGCCGGTGATAAACCGGAGGAAGGTGGGGATGACGTCAAGTCATCATGGCCCTTACGAGTAGGGCTACACACGTGCTACAATGGCGCATACAAAGAGAAGCGACCTCGCGAGAGCAAGCGGACCTCACAAAGTGCGTCGTAGTCCGGATCGGAGTCTGCAACTCGACTCCGTGAAGTCGGAATCGCTAGTAATCGTGGATCAGAATGCCACGGTGAATACGTTCCCGGGCCTTGTACACACCGCCCGTCACACCATGGGAGTGGGTTGCAAAAGAAGTAGGTAGCTTAAC

**Δ*Hfq*-1d-1:**

GCACAGAGGAGCTTGCTCCTTGGGTGACGAGTGGCGGACGGGTGAGTAATGTCTGGGGATCTGCCCGATAGAGGGGGATAACCACTGGAAACGGTGGCTAATACCGCATAACGTCGCAAGACCAAAGAGGGGGACCTTCGGGCCTCTCACTATCGGATGAACCCAGATGGGATTAGCTAGTAGGCGGGGTAATGGCCCACCTAGGCGACGATCCCTAGCTGGTCTGAGAGGATGACCAGCCACACTGGAACTGAGACACGGTCCAGACTCCTACGGGAGGCAGCAGTGGGGAATATTGCACAATGGGCGCAAGCCTGATGCAGCCATGCCGCGTGTATGAAGAAGGCCTTCGGGTTGTAAAGTACTTTCAGCGGGGAGGAAGGCGATGTGGTTAATAACCGCGTCGATTGACGTTACCCGCAGAAGAAGCACCGGCTAACTCCGTGCCAGCAGCCGCGGTAATACGGAGGGTGCAAGCGTTAATCGGAATTACTGGGCGTAAAGCGCACGCAGGCGGTCTGTTAAGTCAGATGTGAAATCCCCGGGCTTAACCTGGGAACTGCATTTGAAACTGGCAGGCTTGAGTCTTGTAGAGGGGGGTAGAATTCCAGGTGTAGCGGTGAAATGCGTAGAGATCTGGAGGAATACCGGTGGCGAAGGCGGCCCCCTGGACAAAGACTGACGCTCAGGTGCGAAAGCGTGGGGAGCAAACAGGATTAGATACCCTGGTAGTCCACGCCGTAAACGATGTCGACTTGGAGGTTGTTCCCTTGAGGAGTGGCTTCCGGAGCTAACGCGTTAAGTCGACCGCCTGGGGAGTACGGCCGCAAGGTTAAAACTCAAATGAATTGACGGGGGCCCGCACAAGCGGTGGAGCATGTGGTTTAATTCGATGCAACGCGAAGAACCTTACCTACTCTTGACATCCAGCGAACTTTCCAGAGATGGATTGGTGCCTTCGGGAACGCTGAGACAGGTGCTGCATGGCTGTCGTCAGCTCGTGTTGTGAAATGTTGGGTTAAGTCCCGCAACGAGCGCAACCCTTATCCTTTGTTGCCAGCGATTCGGTCGGGAACTCAAAGGAGACTGCCGGTGATAAACCGGAGGAAGGTGGGGATGACGTCAAGTCATCATGGCCCTTACGAGTAGGGCTACACACGTGCTACAATGGCGCATACAAAGAGAAGCGACCTCGCGAGAGCAAGCGGACCTCACAAAGTGCGTCGTAGTCCGGATCGGAGTCTGCAACTCGACTCCGTGAAGTCGGAATCGCTAGTAATCGTGGATCAGAATGCCACGGTGAATACGTTCCCGGGCCTTGTACACACCGCCCGTCACACCATGGGAGTGGGTTGCAAAAGAAGTAGGTAGCTTAACCTT

**Δ*Hfq*-1d-2:**

GGTAGCACAGAGGAGCTTGCTCCTTGGGTGACGAGTGGCGGACGGGTGAGTAATGTCTGGGGATCTGCCCGATAGAGGGGGATAACCACTGGAAACGGTGGCTAATACCGCATAACGTCGCAAGACCAAAGAGGGGGACCTTCGGGCCTCTCACTATCGGATGAACCCAGATGGGATTAGCTAGTAGGCGGGGTAATGGCCCACCTAGGCGACGATCCCTAGCTGGTCTGAGAGGATGACCAGCCACACTGGAACTGAGACACGGTCCAGACTCCTACGGGAGGCAGCAGTGGGGAATATTGCACAATGGGCGCAAGCCTGATGCAGCCATGCCGCGTGTATGAAGAAGGCCTTCGGGTTGTAAAGTACTTTCAGCGGGGAGGAAGGCGATGTGGTTAATAACCGCGTCGATTGACGTTACCCGCAGAAGAAGCACCGGCTAACTCCGTGCCAGCAGCCGCGGTAATACGGAGGGTGCAAGCGTTAATCGGAATTACTGGGCGTAAAGCGCACGCAGGCGGTCTGTTAAGTCAGATGTGAAATCCCCGGGCTTAACCTGGGAACTGCATTTGAAACTGGCAGGCTTGAGTCTTGTAGAGGGGGGTAGAATTCCAGGTGTAGCGGTGAAATGCGTAGAGATCTGGAGGAATACCGGTGGCGAAGGCGGCCCCCTGGACAAAGACTGACGCTCAGGTGCGAAAGCGTGGGGAGCAAACAGGATTAGATACCCTGGTAGTCCACGCCGTAAACGATGTCGACTTGGAGGTTGTTCCCTTGAGGAGTGGCTTCCGGAGCTAACGCGTTAAGTCGACCGCCTGGGGAGTACGGCCGCAAGGTTAAAACTCAAATGAATTGACGGGGGCCCGCACAAGCGGTGGAGCATGTGGTTTAATTCGATGCAACGCGAAGAACCTTACCTACTCTTGACATCCAGCGAACTTTCCAGAGATGGATTGGTGCCTTCGGGAACGCTGAGACAGGTGCTGCATGGCTGTCGTCAGCTCGTGTTGTGAAATGTTGGGTTAAGTCCCGCAACGAGCGCAACCCTTATCCTTTGTTGCCAGCGATTCGGTCGGGAACTCAAAGGAGACTGCCGGTGATAAACCGGAGGAAGGTGGGGATGACGTCAAGTCATCATGGCCCTTACGAGTAGGGCTACACACGTGCTACAATGGCGCATACAAAGAGAAGCGACCTCGCGAGAGCAAGCGGACCTCACAAAGTGCGTCGTAGTCCGGATCGGAGTCTGCAACTCGACTCCGTGAAGTCGGAATCGCTAGTAATCGTGGATCAGAATGCCACGGTGAATACGTTCCCGGGCCTTGTACACACCGCCCGTCACACCATGGGAGTGGGTTGCAAAAGAAGTAGGTAGCTTAACCTTCGG

**Δ*Hfq*-1d-3:**

TAGCACAGAGGAGCTTGCTCCTTGGGTGACGAGTGGCGGACGGGTGAGTAATGTCTGGGGATCTGCCCGATAGAGGGGGATAACCACTGGAAACGGTGGCTAATACCGCATAACGTCGCAAGACCAAAGAGGGGGACCTTCGGGCCTCTCACTATCGGATGAACCCAGATGGGATTAGCTAGTAGGCGGGGTAATGGCCCACCTAGGCGACGATCCCTAGCTGGTCTGAGAGGATGACCAGCCACACTGGAACTGAGACACGGTCCAGACTCCTACGGGAGGCAGCAGTGGGGAATATTGCACAATGGGCGCAAGCCTGATGCAGCCATGCCGCGTGTATGAAGAAGGCCTTCGGGTTGTAAAGTACTTTCAGCGGGGAGGAAGGCGATGTGGTTAATAACCGCGTCGATTGACGTTACCCGCAGAAGAAGCACCGGCTAACTCCGTGCCAGCAGCCGCGGTAATACGGAGGGTGCAAGCGTTAATCGGAATTACTGGGCGTAAAGCGCACGCAGGCGGTCTGTTAAGTCAGATGTGAAATCCCCGGGCTTAACCTGGGAACTGCATTTGAAACTGGCAGGCTTGAGTCTTGTAGAGGGGGGTAGAATTCCAGGTGTAGCGGTGAAATGCGTAGAGATCTGGAGGAATACCGGTGGCGAAGGCGGCCCCCTGGACAAAGACTGACGCTCAGGTGCGAAAGCGTGGGGAGCAAACAGGATTAGATACCCTGGTAGTCCACGCCGTAAACGATGTCGACTTGGAGGTTGTTCCCTTGAGGAGTGGCTTCCGGAGCTAACGCGTTAAGTCGACCGCCTGGGGAGTACGGCCGCAAGGTTAAAACTCAAATGAATTGACGGGGGCCCGCACAAGCGGTGGAGCATGTGGTTTAATTCGATGCAACGCGAAGAACCTTACCTACTCTTGACATCCAGCGAACTTTCCAGAGATGGATTGGTGCCTTCGGGAACGCTGAGACAGGTGCTGCATGGCTGTCGTCAGCTCGTGTTGTGAAATGTTGGGTTAAGTCCCGCAACGAGCGCAACCCTTATCCTTTGTTGCCAGCGATTCGGTCGGGAACTCAAAGGAGACTGCCGGTGATAAACCGGAGGAAGGTGGGGATGACGTCAAGTCATCATGGCCCTTACGAGTAGGGCTACACACGTGCTACAATGGCGCATACAAAGAGAAGCGACCTCGCGAGAGCAAGCGGACCTCACAAAGTGCGTCGTAGTCCGGATCGGAGTCTGCAACTCGACTCCGTGAAGTCGGAATCGCTAGTAATCGTGGATCAGAATGCCACGGTGAATACGTTCCCGGGCCTTGTACACACCGCCCGTCACACCATGGGAGTGGGTTGCAAAAGAAGTAGGTAGCTTAACCTTC

**WT-3d-1:**

CACAGAGGAGCTTGCTCCTTGGGTGACGAGTGGCGGACGGGTGAGTAATGTCTGGGGATCTGCCCGATAGAGGGGGATAACCACTGGAAACGGTGGCTAATACCGCATAACGTCGCAAGACCAAAGAGGGGGACCTTCGGGCCTCTCACTATCGGATGAACCCAGATGGGATTAGCTAGTAGGCGGGGTAATGGCCCACCTAGGCGACGATCCCTAGCTGGTCTGAGAGGATGACCAGCCACACTGGAACTGAGACACGGTCCAGACTCCTACGGGAGGCAGCAGTGGGGAATATTGCACAATGGGCGCAAGCCTGATGCAGCCATGCCGCGTGTATGAAGAAGGCCTTCGGGTTGTAAAGTACTTTCAGCGGGGAGGAAGGCGATGTGGTTAATAACCGCGTCGATTGACGTTACCCGCAGAAGAAGCACCGGCTAACTCCGTGCCAGCAGCCGCGGTAATACGGAGGGTGCAAGCGTTAATCGGAATTACTGGGCGTAAAGCGCACGCAGGCGGTCTGTTAAGTCAGATGTGAAATCCCCGGGCTTAACCTGGGAACTGCATTTGAAACTGGCAGGCTTGAGTCTTGTAGAGGGGGGTAGAATTCCAGGTGTAGCGGTGAAATGCGTAGAGATCTGGAGGAATACCGGTGGCGAAGGCGGCCCCCTGGACAAAGACTGACGCTCAGGTGCGAAAGCGTGGGGAGCAAACAGGATTAGATACCCTGGTAGTCCACGCCGTAAACGATGTCGACTTGGAGGTTGTTCCCTTGAGGAGTGGCTTCCGGAGCTAACGCGTTAAGTCGACCGCCTGGGGAGTACGGCCGCAAGGTTAAAACTCAAATGAATTGACGGGGGCCCGCACAAGCGGTGGAGCATGTGGTTTAATTCGATGCAACGCGAAGAACCTTACCTACTCTTGACATCCAGCGAACTTTCCAGAGATGGATTGGTGCCTTCGGGAACGCTGAGACAGGTGCTGCATGGCTGTCGTCAGCTCGTGTTGTGAAATGTTGGGTTAAGTCCCGCAACGAGCGCAACCCTTATCCTTTGTTGCCAGCGATTCGGTCGGGAACTCAAAGGAGACTGCCGGTGATAAACCGGAGGAAGGTGGGGATGACGTCAAGTCATCATGGCCCTTACGAGTAGGGCTACACACGTGCTACAATGGCGCATACAAAGAGAAGCGACCTCGCGAGAGCAAGCGGACCTCACAAAGTGCGTCGTAGTCCGGATCGGAGTCTGCAACTCGACTCCGTGAAGTCGGAATCGCTAGTAATCGTGGATCAGAATGCCACGGTGAATACGTTCCCGGGCCTTGTACACACCGCCCGTCACACCATGGGAGTGGGTTGCAAAAGAAGTAG

**WT-3d-2:**

CACAGAGGAGCTTGCTCCTTGGGTGACGAGTGGCGGACGGGTGAGTAATGTCTGGGGATCTGCCCGATAGAGGGGGATAACCACTGGAAACGGTGGCTAATACCGCATAACGTCGCAAGACCAAAGAGGGGGACCTTCGGGCCTCTCACTATCGGATGAACCCAGATGGGATTAGCTAGTAGGCGGGGTAATGGCCCACCTAGGCGACGATCCCTAGCTGGTCTGAGAGGATGACCAGCCACACTGGAACTGAGACACGGTCCAGACTCCTACGGGAGGCAGCAGTGGGGAATATTGCACAATGGGCGCAAGCCTGATGCAGCCATGCCGCGTGTATGAAGAAGGCCTTCGGGTTGTAAAGTACTTTCAGCGGGGAGGAAGGCGATGTGGTTAATAACCGCGTCGATTGACGTTACCCGCAGAAGAAGCACCGGCTAACTCCGTGCCAGCAGCCGCGGTAATACGGAGGGTGCAAGCGTTAATCGGAATTACTGGGCGTAAAGCGCACGCAGGCGGTCTGTTAAGTCAGATGTGAAATCCCCGGGCTTAACCTGGGAACTGCATTTGAAACTGGCAGGCTTGAGTCTTGTAGAGGGGGGTAGAATTCCAGGTGTAGCGGTGAAATGCGTAGAGATCTGGAGGAATACCGGTGGCGAAGGCGGCCCCCTGGACAAAGACTGACGCTCAGGTGCGAAAGCGTGGGGAGCAAACAGGATTAGATACCCTGGTAGTCCACGCCGTAAACGATGTCGACTTGGAGGTTGTTCCCTTGAGGAGTGGCTTCCGGAGCTAACGCGTTAAGTCGACCGCCTGGGGAGTACGGCCGCAAGGTTAAAACTCAAATGAATTGACGGGGGCCCGCACAAGCGGTGGAGCATGTGGTTTAATTCGATGCAACGCGAAGAACCTTACCTACTCTTGACATCCAGCGAACTTTCCAGAGATGGATTGGTGCCTTCGGGAACGCTGAGACAGGTGCTGCATGGCTGTCGTCAGCTCGTGTTGTGAAATGTTGGGTTAAGTCCCGCAACGAGCGCAACCCTTATCCTTTGTTGCCAGCGATTCGGTCGGGAACTCAAAGGAGACTGCCGGTGATAAACCGGAGGAAGGTGGGGATGACGTCAAGTCATCATGGCCCTTACGAGTAGGGCTACACACGTGCTACAATGGCGCATACAAAGAGAAGCGACCTCGCGAGAGCAAGCGGACCTCACAAAGTGCGTCGTAGTCCGGATCGGAGTCTGCAACTCGACTCCGTGAAGTCGGAATCGCTAGTAATCGTGGATCAGAATGCCACGGTGAATACGTTCCCGGGCCTTGTACACACCGCCCGTCACACCATGGGAGTGGGTTGCAAAAGAAGT

**WT-3d-3:**

GTAGCACAGAGGAGCTTGCTCCTTGGGTGACGAGTGGCGGACGGGTGAGTAATGTCTGGGGATCTGCCCGATAGAGGGGGATAACCACTGGAAACGGTGGCTAATACCGCATAACGTCGCAAGACCAAAGAGGGGGACCTTCGGGCCTCTCACTATCGGATGAACCCAGATGGGATTAGCTAGTAGGCGGGGTAATGGCCCACCTAGGCGACGATCCCTAGCTGGTCTGAGAGGATGACCAGCCACACTGGAACTGAGACACGGTCCAGACTCCTACGGGAGGCAGCAGTGGGGAATATTGCACAATGGGCGCAAGCCTGATGCAGCCATGCCGCGTGTATGAAGAAGGCCTTCGGGTTGTAAAGTACTTTCAGCGGGGAGGAAGGCGATGTGGTTAATAACCGCGTCGATTGACGTTACCCGCAGAAGAAGCACCGGCTAACTCCGTGCCAGCAGCCGCGGTAATACGGAGGGTGCAAGCGTTAATCGGAATTACTGGGCGTAAAGCGCACGCAGGCGGTCTGTTAAGTCAGATGTGAAATCCCCGGGCTTAACCTGGGAACTGCATTTGAAACTGGCAGGCTTGAGTCTTGTAGAGGGGGGTAGAATTCCAGGTGTAGCGGTGAAATGCGTAGAGATCTGGAGGAATACCGGTGGCGAAGGCGGCCCCCTGGACAAAGACTGACGCTCAGGTGCGAAAGCGTGGGGAGCAAACAGGATTAGATACCCTGGTAGTCCACGCCGTAAACGATGTCGACTTGGAGGTTGTTCCCTTGAGGAGTGGCTTCCGGAGCTAACGCGTTAAGTCGACCGCCTGGGGAGTACGGCCGCAAGGTTAAAACTCAAATGAATTGACGGGGGCCCGCACAAGCGGTGGAGCATGTGGTTTAATTCGATGCAACGCGAAGAACCTTACCTACTCTTGACATCCAGCGAACTTTCCAGAGATGGATTGGTGCCTTCGGGAACGCTGAGACAGGTGCTGCATGGCTGTCGTCAGCTCGTGTTGTGAAATGTTGGGTTAAGTCCCGCAACGAGCGCAACCCTTATCCTTTGTTGCCAGCGATTCGGTCGGGAACTCAAAGGAGACTGCCGGTGATAAACCGGAGGAAGGTGGGGATGACGTCAAGTCATCATGGCCCTTACGAGTAGGGCTACACACGTGCTACAATGGCGCATACAAAGAGAAGCGACCTCGCGAGAGCAAGCGGACCTCACAAAGTGCGTCGTAGTCCGGATCGGAGTCTGCAACTCGACTCCGTGAAGTCGGAATCGCTAGTAATCGTGGATCAGAATGCCACGGTGAATACGTTCCCGGGCCTTGTACACACCGCCCGTCACACCATGGGAGTGGGTTGCAAAAGAAGT

**Δ*Hfq*-3d-1:**

CACAGAGGAGCTTGCTCCTTGGGTGACGAGTGGCGGACGGGTGAGTAATGTCTGGGGATCTGCCCGATAGAGGGGGATAACCACTGGAAACGGTGGCTAATACCGCATAACGTCGCAAGACCAAAGAGGGGGACCTTCGGGCCTCTCACTATCGGATGAACCCAGATGGGATTAGCTAGTAGGCGGGGTAATGGCCCACCTAGGCGACGATCCCTAGCTGGTCTGAGAGGATGACCAGCCACACTGGAACTGAGACACGGTCCAGACTCCTACGGGAGGCAGCAGTGGGGAATATTGCACAATGGGCGCAAGCCTGATGCAGCCATGCCGCGTGTATGAAGAAGGCCTTCGGGTTGTAAAGTACTTTCAGCGGGGAGGAAGGCGATGTGGTTAATAACCGCGTCGATTGACGTTACCCGCAGAAGAAGCACCGGCTAACTCCGTGCCAGCAGCCGCGGTAATACGGAGGGTGCAAGCGTTAATCGGAATTACTGGGCGTAAAGCGCACGCAGGCGGTCTGTTAAGTCAGATGTGAAATCCCCGGGCTTAACCTGGGAACTGCATTTGAAACTGGCAGGCTTGAGTCTTGTAGAGGGGGGTAGAATTCCAGGTGTAGCGGTGAAATGCGTAGAGATCTGGAGGAATACCGGTGGCGAAGGCGGCCCCCTGGACAAAGACTGACGCTCAGGTGCGAAAGCGTGGGGAGCAAACAGGATTAGATACCCTGGTAGTCCACGCCGTAAACGATGTCGACTTGGAGGTTGTTCCCTTGAGGAGTGGCTTCCGGAGCTAACGCGTTAAGTCGACCGCCTGGGGAGTACGGCCGCAAGGTTAAAACTCAAATGAATTGACGGGGGCCCGCACAAGCGGTGGAGCATGTGGTTTAATTCGATGCAACGCGAAGAACCTTACCTACTCTTGACATCCAGCGAACTTTCCAGAGATGGATTGGTGCCTTCGGGAACGCTGAGACAGGTGCTGCATGGCTGTCGTCAGCTCGTGTTGTGAAATGTTGGGTTAAGTCCCGCAACGAGCGCAACCCTTATCCTTTGTTGCCAGCGATTCGGTCGGGAACTCAAAGGAGACTGCCGGTGATAAACCGGAGGAAGGTGGGGATGACGTCAAGTCATCATGGCCCTTACGAGTAGGGCTACACACGTGCTACAATGGCGCATACAAAGAGAAGCGACCTCGCGAGAGCAAGCGGACCTCACAAAGTGCGTCGTAGTCCGGATCGGAGTCTGCAACTCGACTCCGTGAAGTCGGAATCGCTAGTAATCGTGGATCAGAATGCCACGGTGAATACGTTCCCGGGCCTTGTACACACCGCCCGTCACACCATGGGAGTGGGTTGCAAAAGAAGT

**Δ*Hfq*-3d-2:**

CACAGAGGAGCTTGCTCCTTGGGTGACGAGTGGCGGACGGGTGAGTAATGTCTGGGGATCTGCCCGATAGAGGGGGATAACCACTGGAAACGGTGGCTAATACCGCATAACGTCGCAAGACCAAAGAGGGGGACCTTCGGGCCTCTCACTATCGGATGAACCCAGATGGGATTAGCTAGTAGGCGGGGTAATGGCCCACCTAGGCGACGATCCCTAGCTGGTCTGAGAGGATGACCAGCCACACTGGAACTGAGACACGGTCCAGACTCCTACGGGAGGCAGCAGTGGGGAATATTGCACAATGGGCGCAAGCCTGATGCAGCCATGCCGCGTGTATGAAGAAGGCCTTCGGGTTGTAAAGTACTTTCAGCGGGGAGGAAGGCGATGTGGTTAATAACCGCGTCGATTGACGTTACCCGCAGAAGAAGCACCGGCTAACTCCGTGCCAGCAGCCGCGGTAATACGGAGGGTGCAAGCGTTAATCGGAATTACTGGGCGTAAAGCGCACGCAGGCGGTCTGTTAAGTCAGATGTGAAATCCCCGGGCTTAACCTGGGAACTGCATTTGAAACTGGCAGGCTTGAGTCTTGTAGAGGGGGGTAGAATTCCAGGTGTAGCGGTGAAATGCGTAGAGATCTGGAGGAATACCGGTGGCGAAGGCGGCCCCCTGGACAAAGACTGACGCTCAGGTGCGAAAGCGTGGGGAGCAAACAGGATTAGATACCCTGGTAGTCCACGCCGTAAACGATGTCGACTTGGAGGTTGTTCCCTTGAGGAGTGGCTTCCGGAGCTAACGCGTTAAGTCGACCGCCTGGGGAGTACGGCCGCAAGGTTAAAACTCAAATGAATTGACGGGGGCCCGCACAAGCGGTGGAGCATGTGGTTTAATTCGATGCAACGCGAAGAACCTTACCTACTCTTGACATCCAGCGAACTTTCCAGAGATGGATTGGTGCCTTCGGGAACGCTGAGACAGGTGCTGCATGGCTGTCGTCAGCTCGTGTTGTGAAATGTTGGGTTAAGTCCCGCAACGAGCGCAACCCTTATCCTTTGTTGCCAGCGATTCGGTCGGGAACTCAAAGGAGACTGCCGGTGATAAACCGGAGGAAGGTGGGGATGACGTCAAGTCATCATGGCCCTTACGAGTAGGGCTACACACGTGCTACAATGGCGCATACAAAGAGAAGCGACCTCGCGAGAGCAAGCGGACCTCACAAAGTGCGTCGTAGTCCGGATCGGAGTCTGCAACTCGACTCCGTGAAGTCGGAATCGCTAGTAATCGTGGATCAGAATGCCACGGTGAATACGTTCCCGGGCCTTGTACACACCGCCCGTCACACCATGGGAGTGGGTTGCAAAAGAAGTAG

**Δ*Hfq*-3d-3:**

GCACAGAGGAGCTTGCTCCTTGGGTGACGAGTGGCGGACGGGTGAGTAATGTCTGGGGATCTGCCCGATAGAGGGGGATAACCACTGGAAACGGTGGCTAATACCGCATAACGTCGCAAGACCAAAGAGGGGGACCTTCGGGCCTCTCACTATCGGATGAACCCAGATGGGATTAGCTAGTAGGCGGGGTAATGGCCCACCTAGGCGACGATCCCTAGCTGGTCTGAGAGGATGACCAGCCACACTGGAACTGAGACACGGTCCAGACTCCTACGGGAGGCAGCAGTGGGGAATATTGCACAATGGGCGCAAGCCTGATGCAGCCATGCCGCGTGTATGAAGAAGGCCTTCGGGTTGTAAAGTACTTTCAGCGGGGAGGAAGGCGATGTGGTTAATAACCGCGTCGATTGACGTTACCCGCAGAAGAAGCACCGGCTAACTCCGTGCCAGCAGCCGCGGTAATACGGAGGGTGCAAGCGTTAATCGGAATTACTGGGCGTAAAGCGCACGCAGGCGGTCTGTTAAGTCAGATGTGAAATCCCCGGGCTTAACCTGGGAACTGCATTTGAAACTGGCAGGCTTGAGTCTTGTAGAGGGGGGTAGAATTCCAGGTGTAGCGGTGAAATGCGTAGAGATCTGGAGGAATACCGGTGGCGAAGGCGGCCCCCTGGACAAAGACTGACGCTCAGGTGCGAAAGCGTGGGGAGCAAACAGGATTAGATACCCTGGTAGTCCACGCCGTAAACGATGTCGACTTGGAGGTTGTTCCCTTGAGGAGTGGCTTCCGGAGCTAACGCGTTAAGTCGACCGCCTGGGGAGTACGGCCGCAAGGTTAAAACTCAAATGAATTGACGGGGGCCCGCACAAGCGGTGGAGCATGTGGTTTAATTCGATGCAACGCGAAGAACCTTACCTACTCTTGACATCCAGCGAACTTTCCAGAGATGGATTGGTGCCTTCGGGAACGCTGAGACAGGTGCTGCATGGCTGTCGTCAGCTCGTGTTGTGAAATGTTGGGTTAAGTCCCGCAACGAGCGCAACCCTTATCCTTTGTTGCCAGCGATTCGGTCGGGAACTCAAAGGAGACTGCCGGTGATAAACCGGAGGAAGGTGGGGATGACGTCAAGTCATCATGGCCCTTACGAGTAGGGCTACACACGTGCTACAATGGCGCATACAAAGAGAAGCGACCTCGCGAGAGCAAGCGGACCTCACAAAGTGCGTCGTAGTCCGGATCGGAGTCTGCAACTCGACTCCGTGAAGTCGGAATCGCTAGTAATCGTGGATCAGAATGCCACGGTGAATACGTTCCCGGGCCTTGTACACACCGCCCGTCACACCATGGGAGTGGGTTGCAAAAGAAGTA

**WT-5d-1:**

GCACAGAGGAGCTTGCTCCTTGGGTGACGAGTGGCGGACGGGTGAGTAATGTCTGGGGATCTGCCCGATAGAGGGGGATAACCACTGGAAACGGTGGCTAATACCGCATAACGTCGCAAGACCAAAGAGGGGGACCTTCGGGCCTCTCACTATCGGATGAACCCAGATGGGATTAGCTAGTAGGCGGGGTAATGGCCCACCTAGGCGACGATCCCTAGCTGGTCTGAGAGGATGACCAGCCACACTGGAACTGAGACACGGTCCAGACTCCTACGGGAGGCAGCAGTGGGGAATATTGCACAATGGGCGCAAGCCTGATGCAGCCATGCCGCGTGTATGAAGAAGGCCTTCGGGTTGTAAAGTACTTTCAGCGGGGAGGAAGGCGATGTGGTTAATAACCGCGTCGATTGACGTTACCCGCAGAAGAAGCACCGGCTAACTCCGTGCCAGCAGCCGCGGTAATACGGAGGGTGCAAGCGTTAATCGGAATTACTGGGCGTAAAGCGCACGCAGGCGGTCTGTTAAGTCAGATGTGAAATCCCCGGGCTTAACCTGGGAACTGCATTTGAAACTGGCAGGCTTGAGTCTTGTAGAGGGGGGTAGAATTCCAGGTGTAGCGGTGAAATGCGTAGAGATCTGGAGGAATACCGGTGGCGAAGGCGGCCCCCTGGACAAAGACTGACGCTCAGGTGCGAAAGCGTGGGGAGCAAACAGGATTAGATACCCTGGTAGTCCACGCCGTAAACGATGTCGACTTGGAGGTTGTTCCCTTGAGGAGTGGCTTCCGGAGCTAACGCGTTAAGTCGACCGCCTGGGGAGTACGGCCGCAAGGTTAAAACTCAAATGAATTGACGGGGGCCCGCACAAGCGGTGGAGCATGTGGTTTAATTCGATGCAACGCGAAGAACCTTACCTACTCTTGACATCCAGCGAACTTTCCAGAGATGGATTGGTGCCTTCGGGAACGCTGAGACAGGTGCTGCATGGCTGTCGTCAGCTCGTGTTGTGAAATGTTGGGTTAAGTCCCGCAACGAGCGCAACCCTTATCCTTTGTTGCCAGCGATTCGGTCGGGAACTCAAAGGAGACTGCCGGTGATAAACCGGAGGAAGGTGGGGATGACGTCAAGTCATCATGGCCCTTACGAGTAGGGCTACACACGTGCTACAATGGCGCATACAAAGAGAAGCGACCTCGCGAGAGCAAGCGGACCTCACAAAGTGCGTCGTAGTCCGGATCGGAGTCTGCAACTCGACTCCGTGAAGTCGGAATCGCTAGTAATCGTGGATCAGAATGCCACGGTGAATACGTTCCCGGGCCTTGTACACACCGCCCGTCACACCATGGGAGTGGGTTGCAAAAGAAGTAGGTAGCTTAACC

**WT-5d-2:**

CACAGAGGAGCTTGCTCCTTGGGTGACGAGTGGCGGACGGGTGAGTAATGTCTGGGGATCTGCCCGATAGAGGGGGATAACCACTGGAAACGGTGGCTAATACCGCATAACGTCGCAAGACCAAAGAGGGGGACCTTCGGGCCTCTCACTATCGGATGAACCCAGATGGGATTAGCTAGTAGGCGGGGTAATGGCCCACCTAGGCGACGATCCCTAGCTGGTCTGAGAGGATGACCAGCCACACTGGAACTGAGACACGGTCCAGACTCCTACGGGAGGCAGCAGTGGGGAATATTGCACAATGGGCGCAAGCCTGATGCAGCCATGCCGCGTGTATGAAGAAGGCCTTCGGGTTGTAAAGTACTTTCAGCGGGGAGGAAGGCGATGTGGTTAATAACCGCGTCGATTGACGTTACCCGCAGAAGAAGCACCGGCTAACTCCGTGCCAGCAGCCGCGGTAATACGGAGGGTGCAAGCGTTAATCGGAATTACTGGGCGTAAAGCGCACGCAGGCGGTCTGTTAAGTCAGATGTGAAATCCCCGGGCTTAACCTGGGAACTGCATTTGAAACTGGCAGGCTTGAGTCTTGTAGAGGGGGGTAGAATTCCAGGTGTAGCGGTGAAATGCGTAGAGATCTGGAGGAATACCGGTGGCGAAGGCGGCCCCCTGGACAAAGACTGACGCTCAGGTGCGAAAGCGTGGGGAGCAAACAGGATTAGATACCCTGGTAGTCCACGCCGTAAACGATGTCGACTTGGAGGTTGTTCCCTTGAGGAGTGGCTTCCGGAGCTAACGCGTTAAGTCGACCGCCTGGGGAGTACGGCCGCAAGGTTAAAACTCAAATGAATTGACGGGGGCCCGCACAAGCGGTGGAGCATGTGGTTTAATTCGATGCAACGCGAAGAACCTTACCTACTCTTGACATCCAGCGAACTTTCCAGAGATGGATTGGTGCCTTCGGGAACGCTGAGACAGGTGCTGCATGGCTGTCGTCAGCTCGTGTTGTGAAATGTTGGGTTAAGTCCCGCAACGAGCGCAACCCTTATCCTTTGTTGCCAGCGATTCGGTCGGGAACTCAAAGGAGACTGCCGGTGATAAACCGGAGGAAGGTGGGGATGACGTCAAGTCATCATGGCCCTTACGAGTAGGGCTACACACGTGCTACAATGGCGCATACAAAGAGAAGCGACCTCGCGAGAGCAAGCGGACCTCACAAAGTGCGTCGTAGTCCGGATCGGAGTCTGCAACTCGACTCCGTGAAGTCGGAATCGCTAGTAATCGTGGATCAGAATGCCACGGTGAATACGTTCCCGGGCCTTGTACACACCGCCCGTCACACCATGGGAGTGGGTTGCAAAAGAAGTAGGTAGCTTA

**WT-5d-3:**

GCACAGAGGAGCTTGCTCCTTGGGTGACGAGTGGCGGACGGGTGAGTAATGTCTGGGGATCTGCCCGATAGAGGGGGATAACCACTGGAAACGGTGGCTAATACCGCATAACGTCGCAAGACCAAAGAGGGGGACCTTCGGGCCTCTCACTATCGGATGAACCCAGATGGGATTAGCTAGTAGGCGGGGTAATGGCCCACCTAGGCGACGATCCCTAGCTGGTCTGAGAGGATGACCAGCCACACTGGAACTGAGACACGGTCCAGACTCCTACGGGAGGCAGCAGTGGGGAATATTGCACAATGGGCGCAAGCCTGATGCAGCCATGCCGCGTGTATGAAGAAGGCCTTCGGGTTGTAAAGTACTTTCAGCGGGGAGGAAGGCGATGTGGTTAATAACCGCGTCGATTGACGTTACCCGCAGAAGAAGCACCGGCTAACTCCGTGCCAGCAGCCGCGGTAATACGGAGGGTGCAAGCGTTAATCGGAATTACTGGGCGTAAAGCGCACGCAGGCGGTCTGTTAAGTCAGATGTGAAATCCCCGGGCTTAACCTGGGAACTGCATTTGAAACTGGCAGGCTTGAGTCTTGTAGAGGGGGGTAGAATTCCAGGTGTAGCGGTGAAATGCGTAGAGATCTGGAGGAATACCGGTGGCGAAGGCGGCCCCCTGGACAAAGACTGACGCTCAGGTGCGAAAGCGTGGGGAGCAAACAGGATTAGATACCCTGGTAGTCCACGCCGTAAACGATGTCGACTTGGAGGTTGTTCCCTTGAGGAGTGGCTTCCGGAGCTAACGCGTTAAGTCGACCGCCTGGGGAGTACGGCCGCAAGGTTAAAACTCAAATGAATTGACGGGGGCCCGCACAAGCGGTGGAGCATGTGGTTTAATTCGATGCAACGCGAAGAACCTTACCTACTCTTGACATCCAGCGAACTTTCCAGAGATGGATTGGTGCCTTCGGGAACGCTGAGACAGGTGCTGCATGGCTGTCGTCAGCTCGTGTTGTGAAATGTTGGGTTAAGTCCCGCAACGAGCGCAACCCTTATCCTTTGTTGCCAGCGATTCGGTCGGGAACTCAAAGGAGACTGCCGGTGATAAACCGGAGGAAGGTGGGGATGACGTCAAGTCATCATGGCCCTTACGAGTAGGGCTACACACGTGCTACAATGGCGCATACAAAGAGAAGCGACCTCGCGAGAGCAAGCGGACCTCACAAAGTGCGTCGTAGTCCGGATCGGAGTCTGCAACTCGACTCCGTGAAGTCGGAATCGCTAGTAATCGTGGATCAGAATGCCACGGTGAATACGTTCCCGGGCCTTGTACACACCGCCCGTCACACCATGGGAGTGGGTTGCAAAAGAAGTAG

**Δ*Hfq*-5d-1:**

GCACAGAGGAGCTTGCTCCTTGGGTGACGAGTGGCGGACGGGTGAGTAATGTCTGGGGATCTGCCCGATAGAGGGGGATAACCACTGGAAACGGTGGCTAATACCGCATAACGTCGCAAGACCAAAGAGGGGGACCTTCGGGCCTCTCACTATCGGATGAACCCAGATGGGATTAGCTAGTAGGCGGGGTAATGGCCCACCTAGGCGACGATCCCTAGCTGGTCTGAGAGGATGACCAGCCACACTGGAACTGAGACACGGTCCAGACTCCTACGGGAGGCAGCAGTGGGGAATATTGCACAATGGGCGCAAGCCTGATGCAGCCATGCCGCGTGTATGAAGAAGGCCTTCGGGTTGTAAAGTACTTTCAGCGGGGAGGAAGGCGATGTGGTTAATAACCGCGTCGATTGACGTTACCCGCAGAAGAAGCACCGGCTAACTCCGTGCCAGCAGCCGCGGTAATACGGAGGGTGCAAGCGTTAATCGGAATTACTGGGCGTAAAGCGCACGCAGGCGGTCTGTTAAGTCAGATGTGAAATCCCCGGGCTTAACCTGGGAACTGCATTTGAAACTGGCAGGCTTGAGTCTTGTAGAGGGGGGTAGAATTCCAGGTGTAGCGGTGAAATGCGTAGAGATCTGGAGGAATACCGGTGGCGAAGGCGGCCCCCTGGACAAAGACTGACGCTCAGGTGCGAAAGCGTGGGGAGCAAACAGGATTAGATACCCTGGTAGTCCACGCCGTAAACGATGTCGACTTGGAGGTTGTTCCCTTGAGGAGTGGCTTCCGGAGCTAACGCGTTAAGTCGACCGCCTGGGGAGTACGGCCGCAAGGTTAAAACTCAAATGAATTGACGGGGGCCCGCACAAGCGGTGGAGCATGTGGTTTAATTCGATGCAACGCGAAGAACCTTACCTACTCTTGACATCCAGCGAACTTTCCAGAGATGGATTGGTGCCTTCGGGAACGCTGAGACAGGTGCTGCATGGCTGTCGTCAGCTCGTGTTGTGAAATGTTGGGTTAAGTCCCGCAACGAGCGCAACCCTTATCCTTTGTTGCCAGCGATTCGGTCGGGAACTCAAAGGAGACTGCCGGTGATAAACCGGAGGAAGGTGGGGATGACGTCAAGTCATCATGGCCCTTACGAGTAGGGCTACACACGTGCTACAATGGCGCATACAAAGAGAAGCGACCTCGCGAGAGCAAGCGGACCTCACAAAGTGCGTCGTAGTCCGGATCGGAGTCTGCAACTCGACTCCGTGAAGTCGGAATCGCTAGTAATCGTGGATCAGAATGCCACGGTGAATACGTTCCCGGGCCTTGTACACACCGCCCGTCACACCATGGGAGTGGGTTGCAAAAGAAGTA

**Δ*Hfq*-5d-2:**

GCACAGAGGAGCTTGCTCCTTGGGTGACGAGTGGCGGACGGGTGAGTAATGTCTGGGGATCTGCCCGATAGAGGGGGATAACCACTGGAAACGGTGGCTAATACCGCATAACGTCGCAAGACCAAAGAGGGGGACCTTCGGGCCTCTCACTATCGGATGAACCCAGATGGGATTAGCTAGTAGGCGGGGTAATGGCCCACCTAGGCGACGATCCCTAGCTGGTCTGAGAGGATGACCAGCCACACTGGAACTGAGACACGGTCCAGACTCCTACGGGAGGCAGCAGTGGGGAATATTGCACAATGGGCGCAAGCCTGATGCAGCCATGCCGCGTGTATGAAGAAGGCCTTCGGGTTGTAAAGTACTTTCAGCGGGGAGGAAGGCGATGTGGTTAATAACCGCGTCGATTGACGTTACCCGCAGAAGAAGCACCGGCTAACTCCGTGCCAGCAGCCGCGGTAATACGGAGGGTGCAAGCGTTAATCGGAATTACTGGGCGTAAAGCGCACGCAGGCGGTCTGTTAAGTCAGATGTGAAATCCCCGGGCTTAACCTGGGAACTGCATTTGAAACTGGCAGGCTTGAGTCTTGTAGAGGGGGGTAGAATTCCAGGTGTAGCGGTGAAATGCGTAGAGATCTGGAGGAATACCGGTGGCGAAGGCGGCCCCCTGGACAAAGACTGACGCTCAGGTGCGAAAGCGTGGGGAGCAAACAGGATTAGATACCCTGGTAGTCCACGCCGTAAACGATGTCGACTTGGAGGTTGTTCCCTTGAGGAGTGGCTTCCGGAGCTAACGCGTTAAGTCGACCGCCTGGGGAGTACGGCCGCAAGGTTAAAACTCAAATGAATTGACGGGGGCCCGCACAAGCGGTGGAGCATGTGGTTTAATTCGATGCAACGCGAAGAACCTTACCTACTCTTGACATCCAGCGAACTTTCCAGAGATGGATTGGTGCCTTCGGGAACGCTGAGACAGGTGCTGCATGGCTGTCGTCAGCTCGTGTTGTGAAATGTTGGGTTAAGTCCCGCAACGAGCGCAACCCTTATCCTTTGTTGCCAGCGATTCGGTCGGGAACTCAAAGGAGACTGCCGGTGATAAACCGGAGGAAGGTGGGGATGACGTCAAGTCATCATGGCCCTTACGAGTAGGGCTACACACGTGCTACAATGGCGCATACAAAGAGAAGCGACCTCGCGAGAGCAAGCGGACCTCACAAAGTGCGTCGTAGTCCGGATCGGAGTCTGCAACTCGACTCCGTGAAGTCGGAATCGCTAGTAATCGTGGATCAGAATGCCACGGTGAATACGTTCCCGGGCCTTGTACACACCGCCCGTCACACCATGGGAGTGGGTTGCAAAAGAAGTAG

**Δ*Hfq*-5d-3:**

CACAGAGGAGCTTGCTCCTTGGGTGACGAGTGGCGGACGGGTGAGTAATGTCTGGGGATCTGCCCGATAGAGGGGGATAACCACTGGAAACGGTGGCTAATACCGCATAACGTCGCAAGACCAAAGAGGGGGACCTTCGGGCCTCTCACTATCGGATGAACCCAGATGGGATTAGCTAGTAGGCGGGGTAATGGCCCACCTAGGCGACGATCCCTAGCTGGTCTGAGAGGATGACCAGCCACACTGGAACTGAGACACGGTCCAGACTCCTACGGGAGGCAGCAGTGGGGAATATTGCACAATGGGCGCAAGCCTGATGCAGCCATGCCGCGTGTATGAAGAAGGCCTTCGGGTTGTAAAGTACTTTCAGCGGGGAGGAAGGCGATGTGGTTAATAACCGCGTCGATTGACGTTACCCGCAGAAGAAGCACCGGCTAACTCCGTGCCAGCAGCCGCGGTAATACGGAGGGTGCAAGCGTTAATCGGAATTACTGGGCGTAAAGCGCACGCAGGCGGTCTGTTAAGTCAGATGTGAAATCCCCGGGCTTAACCTGGGAACTGCATTTGAAACTGGCAGGCTTGAGTCTTGTAGAGGGGGGTAGAATTCCAGGTGTAGCGGTGAAATGCGTAGAGATCTGGAGGAATACCGGTGGCGAAGGCGGCCCCCTGGACAAAGACTGACGCTCAGGTGCGAAAGCGTGGGGAGCAAACAGGATTAGATACCCTGGTAGTCCACGCCGTAAACGATGTCGACTTGGAGGTTGTTCCCTTGAGGAGTGGCTTCCGGAGCTAACGCGTTAAGTCGACCGCCTGGGGAGTACGGCCGCAAGGTTAAAACTCAAATGAATTGACGGGGGCCCGCACAAGCGGTGGAGCATGTGGTTTAATTCGATGCAACGCGAAGAACCTTACCTACTCTTGACATCCAGCGAACTTTCCAGAGATGGATTGGTGCCTTCGGGAACGCTGAGACAGGTGCTGCATGGCTGTCGTCAGCTCGTGTTGTGAAATGTTGGGTTAAGTCCCGCAACGAGCGCAACCCTTATCCTTTGTTGCCAGCGATTCGGTCGGGAACTCAAAGGAGACTGCCGGTGATAAACCGGAGGAAGGTGGGGATGACGTCAAGTCATCATGGCCCTTACGAGTAGGGCTACACACGTGCTACAATGGCGCATACAAAGAGAAGCGACCTCGCGAGAGCAAGCGGACCTCACAAAGTGCGTCGTAGTCCGGATCGGAGTCTGCAACTCGACTCCGTGAAGTCGGAATCGCTAGTAATCGTGGATCAGAATGCCACGGTGAATACGTTCCCGGGCCTTGTACACACCGCCCGTCACACCATGGGAGTGGGTTGCAAAAGAAGTA

**WT-7d-1:**

GCACAGAGGAGCTTGCTCCTTGGGTGACGAGTGGCGGACGGGTGAGTAATGTCTGGGGATCTGCCCGATAGAGGGGGATAACCACTGGAAACGGTGGCTAATACCGCATAACGTCGCAAGACCAAAGAGGGGGACCTTCGGGCCTCTCACTATCGGATGAACCCAGATGGGATTAGCTAGTAGGCGGGGTAATGGCCCACCTAGGCGACGATCCCTAGCTGGTCTGAGAGGATGACCAGCCACACTGGAACTGAGACACGGTCCAGACTCCTACGGGAGGCAGCAGTGGGGAATATTGCACAATGGGCGCAAGCCTGATGCAGCCATGCCGCGTGTATGAAGAAGGCCTTCGGGTTGTAAAGTACTTTCAGCGGGGAGGAAGGCGATGTGGTTAATAACCGCGTCGATTGACGTTACCCGCAGAAGAAGCACCGGCTAACTCCGTGCCAGCAGCCGCGGTAATACGGAGGGTGCAAGCGTTAATCGGAATTACTGGGCGTAAAGCGCACGCAGGCGGTCTGTTAAGTCAGATGTGAAATCCCCGGGCTTAACCTGGGAACTGCATTTGAAACTGGCAGGCTTGAGTCTTGTAGAGGGGGGTAGAATTCCAGGTGTAGCGGTGAAATGCGTAGAGATCTGGAGGAATACCGGTGGCGAAGGCGGCCCCCTGGACAAAGACTGACGCTCAGGTGCGAAAGCGTGGGGAGCAAACAGGATTAGATACCCTGGTAGTCCACGCCGTAAACGATGTCGACTTGGAGGTTGTTCCCTTGAGGAGTGGCTTCCGGAGCTAACGCGTTAAGTCGACCGCCTGGGGAGTACGGCCGCAAGGTTAAAACTCAAATGAATTGACGGGGGCCCGCACAAGCGGTGGAGCATGTGGTTTAATTCGATGCAACGCGAAGAACCTTACCTACTCTTGACATCCAGCGAACTTTCCAGAGATGGATTGGTGCCTTCGGGAACGCTGAGACAGGTGCTGCATGGCTGTCGTCAGCTCGTGTTGTGAAATGTTGGGTTAAGTCCCGCAACGAGCGCAACCCTTATCCTTTGTTGCCAGCGATTCGGTCGGGAACTCAAAGGAGACTGCCGGTGATAAACCGGAGGAAGGTGGGGATGACGTCAAGTCATCATGGCCCTTACGAGTAGGGCTACACACGTGCTACAATGGCGCATACAAAGAGAAGCGACCTCGCGAGAGCAAGCGGACCTCACAAAGTGCGTCGTAGTCCGGATCGGAGTCTGCAACTCGACTCCGTGAAGTCGGAATCGCTAGTAATCGTGGATCAGAATGCCACGGTGAATACGTTCCCGGGCCTTGTACACACCGCCCGTCACACCATGGGAGTGGGTTGCAAAAGAAGT

**WT-7d-2:**

CACAGAGGAGCTTGCTCCTTGGGTGACGAGTGGCGGACGGGTGAGTAATGTCTGGGGATCTGCCCGATAGAGGGGGATAACCACTGGAAACGGTGGCTAATACCGCATAACGTCGCAAGACCAAAGAGGGGGACCTTCGGGCCTCTCACTATCGGATGAACCCAGATGGGATTAGCTAGTAGGCGGGGTAATGGCCCACCTAGGCGACGATCCCTAGCTGGTCTGAGAGGATGACCAGCCACACTGGAACTGAGACACGGTCCAGACTCCTACGGGAGGCAGCAGTGGGGAATATTGCACAATGGGCGCAAGCCTGATGCAGCCATGCCGCGTGTATGAAGAAGGCCTTCGGGTTGTAAAGTACTTTCAGCGGGGAGGAAGGCGATGTGGTTAATAACCGCGTCGATTGACGTTACCCGCAGAAGAAGCACCGGCTAACTCCGTGCCAGCAGCCGCGGTAATACGGAGGGTGCAAGCGTTAATCGGAATTACTGGGCGTAAAGCGCACGCAGGCGGTCTGTTAAGTCAGATGTGAAATCCCCGGGCTTAACCTGGGAACTGCATTTGAAACTGGCAGGCTTGAGTCTTGTAGAGGGGGGTAGAATTCCAGGTGTAGCGGTGAAATGCGTAGAGATCTGGAGGAATACCGGTGGCGAAGGCGGCCCCCTGGACAAAGACTGACGCTCAGGTGCGAAAGCGTGGGGAGCAAACAGGATTAGATACCCTGGTAGTCCACGCCGTAAACGATGTCGACTTGGAGGTTGTTCCCTTGAGGAGTGGCTTCCGGAGCTAACGCGTTAAGTCGACCGCCTGGGGAGTACGGCCGCAAGGTTAAAACTCAAATGAATTGACGGGGGCCCGCACAAGCGGTGGAGCATGTGGTTTAATTCGATGCAACGCGAAGAACCTTACCTACTCTTGACATCCAGCGAACTTTCCAGAGATGGATTGGTGCCTTCGGGAACGCTGAGACAGGTGCTGCATGGCTGTCGTCAGCTCGTGTTGTGAAATGTTGGGTTAAGTCCCGCAACGAGCGCAACCCTTATCCTTTGTTGCCAGCGATTCGGTCGGGAACTCAAAGGAGACTGCCGGTGATAAACCGGAGGAAGGTGGGGATGACGTCAAGTCATCATGGCCCTTACGAGTAGGGCTACACACGTGCTACAATGGCGCATACAAAGAGAAGCGACCTCGCGAGAGCAAGCGGACCTCACAAAGTGCGTCGTAGTCCGGATCGGAGTCTGCAACTCGACTCCGTGAAGTCGGAATCGCTAGTAATCGTGGATCAGAATGCCACGGTGAATACGTTCCCGGGCCTTGTACACACCGCCCGTCACACCATGGGAGTGGGTTGCAAAAGAAGTA

**WT-7d-3:**

CACAGAGGAGCTTGCTCCTTGGGTGACGAGTGGCGGACGGGTGAGTAATGTCTGGGGATCTGCCCGATAGAGGGGGATAACCACTGGAAACGGTGGCTAATACCGCATAACGTCGCAAGACCAAAGAGGGGGACCTTCGGGCCTCTCACTATCGGATGAACCCAGATGGGATTAGCTAGTAGGCGGGGTAATGGCCCACCTAGGCGACGATCCCTAGCTGGTCTGAGAGGATGACCAGCCACACTGGAACTGAGACACGGTCCAGACTCCTACGGGAGGCAGCAGTGGGGAATATTGCACAATGGGCGCAAGCCTGATGCAGCCATGCCGCGTGTATGAAGAAGGCCTTCGGGTTGTAAAGTACTTTCAGCGGGGAGGAAGGCGATGTGGTTAATAACCGCGTCGATTGACGTTACCCGCAGAAGAAGCACCGGCTAACTCCGTGCCAGCAGCCGCGGTAATACGGAGGGTGCAAGCGTTAATCGGAATTACTGGGCGTAAAGCGCACGCAGGCGGTCTGTTAAGTCAGATGTGAAATCCCCGGGCTTAACCTGGGAACTGCATTTGAAACTGGCAGGCTTGAGTCTTGTAGAGGGGGGTAGAATTCCAGGTGTAGCGGTGAAATGCGTAGAGATCTGGAGGAATACCGGTGGCGAAGGCGGCCCCCTGGACAAAGACTGACGCTCAGGTGCGAAAGCGTGGGGAGCAAACAGGATTAGATACCCTGGTAGTCCACGCCGTAAACGATGTCGACTTGGAGGTTGTTCCCTTGAGGAGTGGCTTCCGGAGCTAACGCGTTAAGTCGACCGCCTGGGGAGTACGGCCGCAAGGTTAAAACTCAAATGAATTGACGGGGGCCCGCACAAGCGGTGGAGCATGTGGTTTAATTCGATGCAACGCGAAGAACCTTACCTACTCTTGACATCCAGCGAACTTTCCAGAGATGGATTGGTGCCTTCGGGAACGCTGAGACAGGTGCTGCATGGCTGTCGTCAGCTCGTGTTGTGAAATGTTGGGTTAAGTCCCGCAACGAGCGCAACCCTTATCCTTTGTTGCCAGCGATTCGGTCGGGAACTCAAAGGAGACTGCCGGTGATAAACCGGAGGAAGGTGGGGATGACGTCAAGTCATCATGGCCCTTACGAGTAGGGCTACACACGTGCTACAATGGCGCATACAAAGAGAAGCGACCTCGCGAGAGCAAGCGGACCTCACAAAGTGCGTCGTAGTCCGGATCGGAGTCTGCAACTCGACTCCGTGAAGTCGGAATCGCTAGTAATCGTGGATCAGAATGCCACGGTGAATACGTTCCCGGGCCTTGTACACACCGCCCGTCACACCATGGGAGTGGGTTGCAAAAGAAGTAG

**Δ*Hfq*-7d-1:**

GCACAGAGGAGCTTGCTCCTTGGGTGACGAGTGGCGGACGGGTGAGTAATGTCTGGGGATCTGCCCGATAGAGGGGGATAACCACTGGAAACGGTGGCTAATACCGCATAACGTCGCAAGACCAAAGAGGGGGACCTTCGGGCCTCTCACTATCGGATGAACCCAGATGGGATTAGCTAGTAGGCGGGGTAATGGCCCACCTAGGCGACGATCCCTAGCTGGTCTGAGAGGATGACCAGCCACACTGGAACTGAGACACGGTCCAGACTCCTACGGGAGGCAGCAGTGGGGAATATTGCACAATGGGCGCAAGCCTGATGCAGCCATGCCGCGTGTATGAAGAAGGCCTTCGGGTTGTAAAGTACTTTCAGCGGGGAGGAAGGCGATGTGGTTAATAACCGCGTCGATTGACGTTACCCGCAGAAGAAGCACCGGCTAACTCCGTGCCAGCAGCCGCGGTAATACGGAGGGTGCAAGCGTTAATCGGAATTACTGGGCGTAAAGCGCACGCAGGCGGTCTGTTAAGTCAGATGTGAAATCCCCGGGCTTAACCTGGGAACTGCATTTGAAACTGGCAGGCTTGAGTCTTGTAGAGGGGGGTAGAATTCCAGGTGTAGCGGTGAAATGCGTAGAGATCTGGAGGAATACCGGTGGCGAAGGCGGCCCCCTGGACAAAGACTGACGCTCAGGTGCGAAAGCGTGGGGAGCAAACAGGATTAGATACCCTGGTAGTCCACGCCGTAAACGATGTCGACTTGGAGGTTGTTCCCTTGAGGAGTGGCTTCCGGAGCTAACGCGTTAAGTCGACCGCCTGGGGAGTACGGCCGCAAGGTTAAAACTCAAATGAATTGACGGGGGCCCGCACAAGCGGTGGAGCATGTGGTTTAATTCGATGCAACGCGAAGAACCTTACCTACTCTTGACATCCAGCGAACTTTCCAGAGATGGATTGGTGCCTTCGGGAACGCTGAGACAGGTGCTGCATGGCTGTCGTCAGCTCGTGTTGTGAAATGTTGGGTTAAGTCCCGCAACGAGCGCAACCCTTATCCTTTGTTGCCAGCGATTCGGTCGGGAACTCAAAGGAGACTGCCGGTGATAAACCGGAGGAAGGTGGGGATGACGTCAAGTCATCATGGCCCTTACGAGTAGGGCTACACACGTGCTACAATGGCGCATACAAAGAGAAGCGACCTCGCGAGAGCAAGCGGACCTCACAAAGTGCGTCGTAGTCCGGATCGGAGTCTGCAACTCGACTCCGTGAAGTCGGAATCGCTAGTAATCGTGGATCAGAATGCCACGGTGAATACGTTCCCGGGCCTTGTACACACCGCCCGTCACACCATGGGAGTGGGTTGCAAAAGAAGTAG

**Δ*Hfq*-7d-2:**

ACAGAGGAGCTTGCTCCTTGGGTGACGAGTGGCGGACGGGTGAGTAATGTCTGGGGATCTGCCCGATAGAGGGGGATAACCACTGGAAACGGTGGCTAATACCGCATAACGTCGCAAGACCAAAGAGGGGGACCTTCGGGCCTCTCACTATCGGATGAACCCAGATGGGATTAGCTAGTAGGCGGGGTAATGGCCCACCTAGGCGACGATCCCTAGCTGGTCTGAGAGGATGACCAGCCACACTGGAACTGAGACACGGTCCAGACTCCTACGGGAGGCAGCAGTGGGGAATATTGCACAATGGGCGCAAGCCTGATGCAGCCATGCCGCGTGTATGAAGAAGGCCTTCGGGTTGTAAAGTACTTTCAGCGGGGAGGAAGGCGATGTGGTTAATAACCGCGTCGATTGACGTTACCCGCAGAAGAAGCACCGGCTAACTCCGTGCCAGCAGCCGCGGTAATACGGAGGGTGCAAGCGTTAATCGGAATTACTGGGCGTAAAGCGCACGCAGGCGGTCTGTTAAGTCAGATGTGAAATCCCCGGGCTTAACCTGGGAACTGCATTTGAAACTGGCAGGCTTGAGTCTTGTAGAGGGGGGTAGAATTCCAGGTGTAGCGGTGAAATGCGTAGAGATCTGGAGGAATACCGGTGGCGAAGGCGGCCCCCTGGACAAAGACTGACGCTCAGGTGCGAAAGCGTGGGGAGCAAACAGGATTAGATACCCTGGTAGTCCACGCCGTAAACGATGTCGACTTGGAGGTTGTTCCCTTGAGGAGTGGCTTCCGGAGCTAACGCGTTAAGTCGACCGCCTGGGGAGTACGGCCGCAAGGTTAAAACTCAAATGAATTGACGGGGGCCCGCACAAGCGGTGGAGCATGTGGTTTAATTCGATGCAACGCGAAGAACCTTACCTACTCTTGACATCCAGCGAACTTTCCAGAGATGGATTGGTGCCTTCGGGAACGCTGAGACAGGTGCTGCATGGCTGTCGTCAGCTCGTGTTGTGAAATGTTGGGTTAAGTCCCGCAACGAGCGCAACCCTTATCCTTTGTTGCCAGCGATTCGGTCGGGAACTCAAAGGAGACTGCCGGTGATAAACCGGAGGAAGGTGGGGATGACGTCAAGTCATCATGGCCCTTACGAGTAGGGCTACACACGTGCTACAATGGCGCATACAAAGAGAAGCGACCTCGCGAGAGCAAGCGGACCTCACAAAGTGCGTCGTAGTCCGGATCGGAGTCTGCAACTCGACTCCGTGAAGTCGGAATCGCTAGTAATCGTGGATCAGAATGCCACGGTGAATACGTTCCCGGGCCTTGTACACACCGCCCGTCACACCATGGGAGTGGGTTGCAAAAGAAGTAG

**Δ*Hfq*-7d-3:**

TAGCACAGAGGAGCTTGCTCCTTGGGTGACGAGTGGCGGACGGGTGAGTAATGTCTGGGGATCTGCCCGATAGAGGGGGATAACCACTGGAAACGGTGGCTAATACCGCATAACGTCGCAAGACCAAAGAGGGGGACCTTCGGGCCTCTCACTATCGGATGAACCCAGATGGGATTAGCTAGTAGGCGGGGTAATGGCCCACCTAGGCGACGATCCCTAGCTGGTCTGAGAGGATGACCAGCCACACTGGAACTGAGACACGGTCCAGACTCCTACGGGAGGCAGCAGTGGGGAATATTGCACAATGGGCGCAAGCCTGATGCAGCCATGCCGCGTGTATGAAGAAGGCCTTCGGGTTGTAAAGTACTTTCAGCGGGGAGGAAGGCGATGTGGTTAATAACCGCGTCGATTGACGTTACCCGCAGAAGAAGCACCGGCTAACTCCGTGCCAGCAGCCGCGGTAATACGGAGGGTGCAAGCGTTAATCGGAATTACTGGGCGTAAAGCGCACGCAGGCGGTCTGTTAAGTCAGATGTGAAATCCCCGGGCTTAACCTGGGAACTGCATTTGAAACTGGCAGGCTTGAGTCTTGTAGAGGGGGGTAGAATTCCAGGTGTAGCGGTGAAATGCGTAGAGATCTGGAGGAATACCGGTGGCGAAGGCGGCCCCCTGGACAAAGACTGACGCTCAGGTGCGAAAGCGTGGGGAGCAAACAGGATTAGATACCCTGGTAGTCCACGCCGTAAACGATGTCGACTTGGAGGTTGTTCCCTTGAGGAGTGGCTTCCGGAGCTAACGCGTTAAGTCGACCGCCTGGGGAGTACGGCCGCAAGGTTAAAACTCAAATGAATTGACGGGGGCCCGCACAAGCGGTGGAGCATGTGGTTTAATTCGATGCAACGCGAAGAACCTTACCTACTCTTGACATCCAGCGAACTTTCCAGAGATGGATTGGTGCCTTCGGGAACGCTGAGACAGGTGCTGCATGGCTGTCGTCAGCTCGTGTTGTGAAATGTTGGGTTAAGTCCCGCAACGAGCGCAACCCTTATCCTTTGTTGCCAGCGATTCGGTCGGGAACTCAAAGGAGACTGCCGGTGATAAACCGGAGGAAGGTGGGGATGACGTCAAGTCATCATGGCCCTTACGAGTAGGGCTACACACGTGCTACAATGGCGCATACAAAGAGAAGCGACCTCGCGAGAGCAAGCGGACCTCACAAAGTGCGTCGTAGTCCGGATCGGAGTCTGCAACTCGACTCCGTGAAGTCGGAATCGCTAGTAATCGTGGATCAGAATGCCACGGTGAATACGTTCCCGGGCCTTGTACACACCGCCCGTCACACCATGGGAGTGGGTTGCAAAAGAAGTA

**
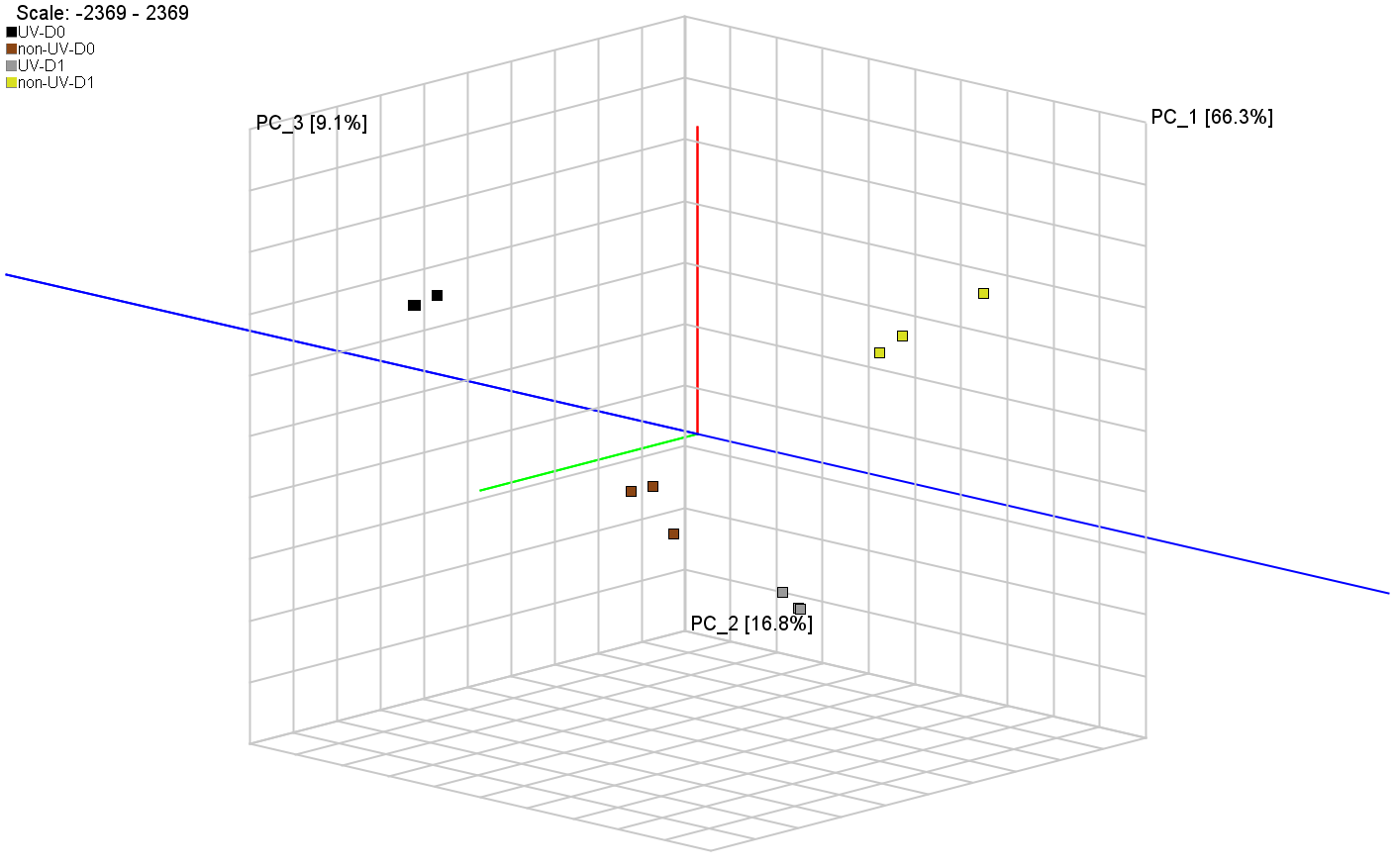
**

**Fig. S1.** PCA score plot of volatile components in samples.

**Table S2** Effect of UV light treatment on TBA values of chicken meat stored at 4℃ for 0 d and 1 d.

| Groups | 0 d | 1 d |
| --- | --- | --- |
| Control group | 0.142±0.004^a^ | 0.167±0.002^a^ |
| UV treated group | 0.143±0.002^a^ | 0.166±0.004^a^ |

Note: Different letters in the same column represent significant differences (p < 0.05).

**Key analytical parameters for GC-IMS and GC-MS**

For GC-IMS, nitrogen was used as the carrier and drift gas, with an IMS temperature of 45°C. RI were calculated using n-alkanones (C₄–C₉), with matching thresholds set at RI ±30 and Dt ±0.02. VOCs were identified by comparing their retention and drift times with the built-in NIST and IMS databases.

For GC-MS, n-alkanes were used as the standard for RI calculation, with a mass scan range of 40 u to 550 u. VOCs were putatively identified by matching their mass spectra with the NIST 17 database (match factor ≥ 80%) and literature RI values. Semi-quantitative analysis was performed using 2-methyl-3-heptanone as the internal standard, with relative contents determined by peak area ratio to the internal standard.

**Table S3** GC-IMS global area set integration parameters obtained from inoculated and non-inoculated chicken samples.

| Compounds | Count | RI | Rt | Dt | Peak volume | | | | | |
| --- | --- | --- | --- | --- | --- | --- | --- | --- | --- | --- |
|  |  |  |  |  | 1d | | | 7d | | |
|  |  |  |  |  | C1 | W1 | K1 | C7 | W7 | K7 |
| Aldehydes | | | | | | | | | | |
| Benzaldehyde | 2 | 1508 | 964 | 1.15 | 51.07±6.03^a^ | 52.12±2.11^a^ | 62.55±6.11^a^ | 87.17±0.65^a^ | 90.44±22.53^a^ | 72.49±8.60^a^ |
| 1-Nonanal | 13 | 1393 | 760 | 1.47 | 62.17±5.75^a^ | 96.05±20.99^a^ | 58.11±9.27^a^ | 68.20±18.08^a^ | 46.32±3.59^a^ | 57.65±3.96^a^ |
| 1-octanal | 14 | 1296 | 606 | 1.40 | 20.41±1.14^a^ | 61.33±24.02^a^ | 20.33±0.90^a^ | 24.33±2.30^a^ | 28.46±4.14^a^ | 29.88±3.47^a^ |
| 3-Methyl-2-butenal | 19 | 1237 | 506 | 1.36 | 16.12±4.58^c^ | 12.01±2.55^c^ | 11.51±1.80^c^ | 277.61±61.49^a^ | 393.49±22.45^a^ | 83.45±14.48^b^ |
| 1-hexanal-M | 36 | 1084 | 303 | 1.26 | 245.82±1.81^a^ | 456.09±105.96^a^ | 165.86±82.41^ab^ | 102.10±13.36^b^ | 164.30±2.13^a^ | 88.94±8.80^b^ |
| 1-hexanal-D | 39 | 1085 | 304 | 1.55 | 16.18±1.21^a^ | 68.25±38.15^a^ | 14.07±7.86^a^ | 8.67±0.84^a^ | 10.68±4.13^a^ | 8.45±2.84^a^ |
| Butanal | 48 | 907 | 173 | 1.29 | 333.70±96.55^b^ | 480.50±72.19^b^ | 349.00±217.92^b^ | 1260.86±17.34^a^ | 1338.75±19.59^a^ | 1330.56±24.73^a^ |
| Alcohols | | | | | | | | | | |
| 1 -hexanol | 11 | 1366 | 714 | 1.32 | 122.86±24.30^a^ | 160.95±12.17^a^ | 94.01±18.66^a^ | 151.73±23.42^a^ | 130.36±1.44^a^ | 153.91±9.41^a^ |
| 1-Pentanol-M | 16 | 1256 | 536 | 1.25 | 459.97±13.84^ab^ | 787.82±113.14^a^ | 418.36±19.03^b^ | 172.08±49.62^cd^ | 118.00±8.22^d^ | 169.84±0.53^c^ |
| 1-Pentanol-D | 17 | 1255 | 535 | 1.50 | 77.68±10.91^a^ | 238.43±77.98^a^ | 67.94±6.81^a^ | 27.06±4.91^a^ | 22.59±1.53^a^ | 29.28±0.90^a^ |
| 1-Butanol, 3-methyl-M | 18 | 1206 | 460 | 1.24 | 476.18±123.08^b^ | 182.02±12.38^b^ | 273.28±55.13^b^ | 1533.95±80.57^a^ | 1434.41±74.97^a^ | 1310.65±57.91^a^ |
| 1-Butanol, 3-methyl-D | 22 | 1207 | 462 | 1.48 | 131.31±34.61^b^ | 100.35±18.98^d^ | 108.31±11.58^c^ | 1636.22±355.21^a^ | 1335.43±480.06^a^ | 1757.11±53.40^a^ |
| 1-butanol-M | 26 | 1142 | 371 | 1.18 | 380.44±70.48^bc^ | 379.14±2.63^bc^ | 373.17±69.67^bc^ | 394.81±4.97^b^ | 547.72±6.64^a^ | 362.06±5.22^c^ |
| 1- butanol-D | 28 | 1141 | 370 | 1.38 | 31.31±9.73^c^ | 33.56±2.08^c^ | 30.80±10.49^c^ | 49.89±2.75^b^ | 90.54±3.38^a^ | 49.31±0.69^b^ |
| 1-Propanol, 2-methyl-M | 32 | 1093 | 312 | 1.17 | 118.67±28.17^b^ | 114.00±11.95^b^ | 96.38±40.27^b^ | 1206.53±91.42^a^ | 1334.75±195.29^a^ | 1330.81±18.08^a^ |
| 1-Propanol, 2-methyl-D | 33 | 1092 | 311 | 1.36 | 37.85±4.94^b^ | 52.46±10.07^b^ | 22.05±12.22^b^ | 468.89±3.29^a^ | 602.66±170.32^a^ | 569.22±77.11^a^ |
| 1-Propanol-M | 34 | 1043 | 267 | 1.12 | 955.95±84.67^c^ | 895.02±61.31^c^ | 861.29±151.70^c^ | 1075.41±98.04^ab^ | 1080.70±16.49^b^ | 1159.43±5.56^a^ |
| 1-Propanol-D | 35 | 1037 | 261 | 1.26 | 583.29±57.27^c^ | 473.84±35.63^c^ | 467.11±86.21^c^ | 1775.19±92.60^ab^ | 1680.48±49.97^b^ | 1869.04±26.06^a^ |
| Ethanol | 46 | 938 | 191 | 1.13 | 14910.12±127.16^a^ | 14498.27±344.95^ab^ | 14478.05±711.78^ab^ | 14061.50±362.03^b^ | 14110.27±18.11^b^ | 15139.58±77.91^a^ |
| Ketones | | | | | | | | | | |
| 2-Butanone, 3-hydroxy-M | 7 | 1295 | 605 | 1.06 | 3666.46±729.70^a^ | 1299.62±204.09^b^ | 3750.21±589.52^a^ | 2486.76±2.63^a^ | 2446.36±375.25^ab^ | 2397.23±47.62^a^ |
| 1 -hydroxy  -2-propanone | 9 | 1294 | 603 | 1.23 | 204.12±95.92^b^ | 29.83±2.24^b^ | 316.593±203.44^ab^ | 664.74±53.33^a^ | 587.32±145.20^ab^ | 674.89±75.45^a^ |
| 2-Butanone, 3-hydroxy-D | 10 | 1294 | 603 | 1.32 | 3286.68±1756.53^ab^ | 175.54±29.77^b^ | 4977.51±3596.27^ab^ | 7981.98±1231.42^a^ | 6715.49±1550.94^a^ | 8484.93±482.82^a^ |
| 2,3-Butanedione | 41 | 1009 | 240 | 1.17 | 3563.42±79.34^a^ | 3215.93±23.45^b^ | 3290.28±57.62^ab^ | 3240.18±7.02^b^ | 2892.60±80.36^c^ | 3079.53±95.25^bc^ |
| 2-Pentanone-M | 44 | 993 | 228 | 1.13 | 1954.29±93.25^a^ | 1804.86±170.62^ab^ | 1890.86±205.60^ab^ | 1571.00±118.99^ab^ | 1543.09±42.51^b^ | 1865.52±68.59^a^ |
| 2-Butanone | 47 | 917 | 179 | 1.24 | 606.28±244.30^c^ | 1063.31±337.55^c^ | 686.39±594.81^c^ | 2216.59±145.00^b^ | 3164.60±178.36^a^ | 3154.61±229.17^a^ |
| 2-Pentanone-D | 50 | 983 | 221 | 1.36 | 51.66±0.28^b^ | 52.80±9.88^b^ | 27.64±3.41^b^ | 257.34±40.59^a^ | 199.62±56.50^ab^ | 188.33±34.23^a^ |
| 2-propanone | 51 | 848 | 143 | 1.12 | 11781.47±145.59^c^ | 12964.93±3.81^a^ | 12455.63±60.41^b^ | 12412.23±239.48^bc^ | 13941.59±409.07^a^ | 12065.65±41.27^b^ |
| Esters | | | | | | | | | | |
| Ethyl heptanoate | 12 | 1341 | 673 | 1.40 | 62.00±19.94^ab^ | 157.07±42.97^a^ | 52.33±3.77^a^ | 19.84±0.79^b^ | 21.39±5.10^b^ | 28.46±1.78^ab^ |
| ethyl (E)-2-butenoate-M | 25 | 1162 | 398 | 1.18 | 26.84±0.72^d^ | 23.50±1.95^d^ | 27.67±3.14^d^ | 135.63±4.72^b^ | 221.11±1.56^a^ | 80.07±2.73^c^ |
| ethyl (E)-2-butenoate-D | 29 | 1161 | 397 | 1.55 | 9.46±2.44^d^ | 10.72±1.21^d^ | 11.47±0.25^d^ | 39.56±6.10^b^ | 92.25±10.45^a^ | 20.54±1.14^c^ |
| Amyl acetate | 30 | 1187 | 433 | 1.30 | 32.32±0.73^a^ | 33.15±7.58^a^ | 34.06±0.85^a^ | 66.03±0.01^a^ | 228.00±108.74^a^ | 44.18±5.19^a^ |
| sec-butyl acetate | 45 | 982 | 220 | 1.22 | 455.69±0.38^b^ | 324.82±30.49^c^ | 371.88±42.15^bc^ | 555.71±12.12^ab^ | 465.49±31.54^b^ | 604.62±12.78^a^ |
| Acetic acid ethyl ester | 49 | 900 | 169 | 1.33 | 1320.39±161.77^b^ | 1440.90±62.16^b^ | 1241.36±201.78^b^ | 3692.16±127.07^a^ | 3245.79±245.76^a^ | 3327.20±90.52^a^ |
| Ethyl propanoate | 54 | 960 | 205 | 1.44 | 9.80±0.90^b^ | 10.01±0.37^b^ | 10.38±0.76^b^ | 325.43±54.30^a^ | 232.89±22.56^a^ | 191.78±2.54^a^ |
| Acids | | | | | | | | | | |
| Acetic acid-M | 1 | 1474 | 897 | 1.05 | 2299.91±51.15^c^ | 2011.69±123.09^c^ | 2740.51±57.53^b^ | 5308.07±273.19^a^ | 5047.39±1214.06^ab^ | 5074.92±179.85^a^ |
| Acetic acid-D | 3 | 1471 | 893 | 1.15 | 90.14±4.64^a^ | 120.20±31.31^a^ | 127.10±15.95^a^ | 722.93±156.26^a^ | 872.11±597.74^a^ | 617.61±79.82^a^ |
| Propanoic acid | 4 | 1559 | 1082 | 1.11 | 109.29±9.99^a^ | 93.94±2.18^a^ | 104.16±4.81^a^ | 276.01±32.14^a^ | 333.01±101.44^a^ | 236.22±16.95^a^ |
| 2-Methyl propanoic acid | 5 | 1596 | 1175 | 1.15 | 104.14±20.82^b^ | 116.66±4.63^b^ | 107.71±11.70^b^ | 398.43±89.41^a^ | 423.69±90.18^a^ | 374.01±57.54^a^ |
| Pyrazines | | | | | | | | | | |
| 2,3-Diethyl-5-methylpyrazine | 6 | 1507 | 963 | 1.28 | 15.28±0.33^b^ | 18.05±0.88^b^ | 15.26±1.94^b^ | 31.20±8.71^a^ | 35.88±4.16^a^ | 28.44±3.74^ab^ |
| Pyrazine | 21 | 1207 | 461 | 1.27 | 242.76±3.39^b^ | 118.75±1.39^d^ | 197.03±4.38^c^ | 2303.85±582.93^a^ | 2826.36±568.41^a^ | 2048.45±90.68^a^ |
| Nitrile | | | | | | | | | | |
| Acrylonitrile | 43 | 1012 | 242 | 1.09 | 1046.26±9.53^b^ | 950.85±22.30^c^ | 1044.07±56.09^bc^ | 1289.23±5.02^a^ | 1159.49±106.66^ab^ | 1274.71±50.98^a^ |
| Amines | | | | | | | | | | |
| Triethylamine | 52 | 800 | 123 | 1.08 | 1486.71±21.99^b^ | 1055.56±15.28^c^ | 1122.07±90.31^c^ | 3156.22±17.05^a^ | 3134.63±239.95^a^ | 3090.55±149.21^a^ |
| Ether | | | | | | | | | | |
| Tetrahydrofuran | 55 | 894 | 166 | 1.22 | 627.32±32.07^b^ | 831.09±6.91^a^ | 696.66±65.66^ab^ | 513.85±2.29^c^ | 843.56±22.65^a^ | 482.13±12.80^c^ |
| Others | | | | | | | | | | |
| 1 | 8 | 1295 | 605 | 1.16 | 2055.32±224.27^b^ | 748.11±93.65^b^ | 2861.80±781.25^b^ | 9655.78±1315.28^a^ | 11165.79±1130.83^a^ | 9314.48±137.07^a^ |
| 2 | 15 | 1312 | 628 | 1.10 | 62.72±1.22^a^ | 82.24±16.90^a^ | 97.73±34.27^a^ | 39.43±9.05^a^ | 64.39±1.70^a^ | 45.72±8.28^a^ |
| 3 | 20 | 1238 | 507 | 1.79 | 27.19±9.18^d^ | 25.20±15.08^d^ | 43.98±43.89^b^ | 37.99±9.67^c^ | 56.00±0.95^a^ | 14.50±3.42^e^ |
| 4 | 23 | 1206 | 460 | 1.78 | 60.86±30.72^b^ | 57.27±41.35^b^ | 114.97±122.69^ab^ | 207.99±62.08^ab^ | 190.24±105.53^ab^ | 291.78±15.31^a^ |
| 5 | 24 | 1187 | 434 | 1.13 | 74.81±11.73^a^ | 38.82±4.50^a^ | 50.59±2.84^a^ | 29.08±5.45^a^ | 128.87±66.46^a^ | 62.09±3.06^a^ |
| 6 | 27 | 1141 | 370 | 1.25 | 105.84±24.70^bc^ | 87.32±1.92^c^ | 78.16±18.70^c^ | 127.63±2.25^b^ | 163.12±10.59^a^ | 152.89±3.70^a^ |
| 7 | 31 | 1198 | 449 | 1.40 | 45.36±4.26^b^ | 35.20±4.32^b^ | 48.71±2.28^b^ | 490.06±66.96^a^ | 372.04±7.72^a^ | 386.84±57.10^a^ |
| 8 | 37 | 1034 | 259 | 1.38 | 20.19±1.11^b^ | 14.04±0.17^b^ | 11.87±4.30^b^ | 204.43±9.33^a^ | 242.58±30.02^a^ | 191.41±4.05^a^ |
| 9 | 38 | 1035 | 260 | 1.55 | 19.00±1.15^a^ | 19.00±1.38^a^ | 20.33±3.25^a^ | 116.08±35.72^a^ | 265.92±128.64^a^ | 81.25±0.20^a^ |
| 10 | 40 | 1035 | 260 | 1.19 | 569.12±31.44^a^ | 518.96±23.25^a^ | 505.44±52.12^a^ | 289.89±2.34^b^ | 218.30±10.95^b^ | 317.44±16.92^b^ |
| 11 | 42 | 1026 | 253 | 1.13 | 114.92±0.54^ab^ | 109.51±5.90^ab^ | 99.00±6.25^b^ | 167.31±1.00^a^ | 158.04±20.53^ab^ | 180.81±23.26^a^ |
| 12 | 53 | 961 | 206 | 1.26 | 61.01±3.16^c^ | 53.98±5.25^c^ | 47.02±6.45^c^ | 265.56±5.34^a^ | 187.81±1.34^b^ | 232.37±12.61^a^ |

Note: Different letters on the same line represented differences between different treatment groups, p < 0.05.

RI: Retention index.

Rt: Retention time in the capillary GC column.

Dt: The drift time in the drift tube.

**Table S4** Similarity between samples (after removing substances with large differences).

|  | non-UV-D0 | non-UV-D0 | non-UV-D0 | UV-D0 | UV-D0 | UV-D0 | non-UV-D1 | non-UV-D1 | non-UV-D1 | UV-D1 | UV-D1 | UV-D1 |
| --- | --- | --- | --- | --- | --- | --- | --- | --- | --- | --- | --- | --- |
| non-UV-D0 | 100 |  |  |  |  |  |  |  |  |  |  |  |
| non-UV-D0 | 97 | 100 |  |  |  |  |  |  |  |  |  |  |
| non-UV-D0 | 96 | 96 | 100 |  |  |  |  |  |  |  |  |  |
| UV-D0 | 88 | 90 | 91 | 100 |  |  |  |  |  |  |  |  |
| UV-D0 | 89 | 90 | 91 | 97 | 100 |  |  |  |  |  |  |  |
| UV-D0 | 89 | 91 | 91 | 99 | 97 | 100 |  |  |  |  |  |  |
| non-UV-D1 | 88 | 89 | 89 | 86 | 86 | 86 | 100 |  |  |  |  |  |
| non-UV-D1 | 90 | 91 | 91 | 87 | 88 | 87 | 97 | 100 |  |  |  |  |
| non-UV-D1 | 90 | 91 | 90 | 87 | 88 | 87 | 97 | 97 | 100 |  |  |  |
| UV-D1 | 92 | 92 | 93 | 86 | 86 | 86 | 91 | 93 | 93 | 100 |  |  |
| UV-D1 | 93 | 93 | 93 | 86 | 86 | 87 | 90 | 92 | 93 | 98 | 100 |  |
| UV-D1 | 93 | 93 | 93 | 87 | 87 | 87 | 91 | 93 | 93 | 98 | 98 | 100 |

**Table S5** Potential Contaminant VOCs.

| Compounds | Molecular formula | Potential source |
| --- | --- | --- |
| Krypton | Kr | Blank detection |
| (CH_3_)_2_NCl | C_2_H_6_ClN | Blank detection |
| 1-benzylindole | C_15_H_13_N | Blank detection |
| N-Methyl-4-pyridinamine | C_6_H_8_N_2_ | Blank detection |
| Amphetamine-3-methyl | C_10_H_15_N | Blank detection |
| 3-Methoxyamphetamine | C_10_H_15_NO | Blank detection |
| Cathine | C_9_H_13_NO | Blank detection |
| Phenol, 4-[2-(methylamino)ethyl]- | C_9_H_13_NO | Blank detection |
| Methane, dichloronitro- | CHCl_2_NO_2_ | Halogenated solvent |
| Benzene, (2-methyloctyl)- | C_15_H_24_ | Plasticizer |
| Cyclotrisiloxane, hexamethyl- | C_6_H_18_O_3_Si_3_ | Column/septum |
| Cyclohexasiloxane, dodecamethyl- | C_12_H_36_O_6_Si_6_ | Column/septum |
| Cyclotetrasiloxane, octamethyl- | C_8_H_24_O_4_Si_4_ | Column/septum |
| Cycloheptasiloxane, tetradecamethyl- | C_14_H_42_O_7_Si_7_ | Column/septum |
| Cyclopentasiloxane, decamethyl- | C_10_H_30_O_5_Si_5_ | Column/septum |
| 1-Octadecanol, TMS derivative | C_21_H_46_OSi | Silylation reagent |
| Phthalic acid, di(3,5-dimethylphenyl) ester | C_24_H_22_O_4_ | Plastic consumables |
| Tetrachloroethylene | C_2_Cl_4_ | Halogenated solvent |
| Methylene chloride | CH_2_Cl_2_ | Halogenated solvent |
| Dimethyl ether | C_2_H_6_O | Carrier gas impurity |
| Terpinen-4-ol | C_10_H_18_O | Plant flavor |
| 6-Nitro-1,3-benzoxazole-2-thiol, TMS | C_10_H_12_N_2_O_3_SSi | Synthetic reagent |


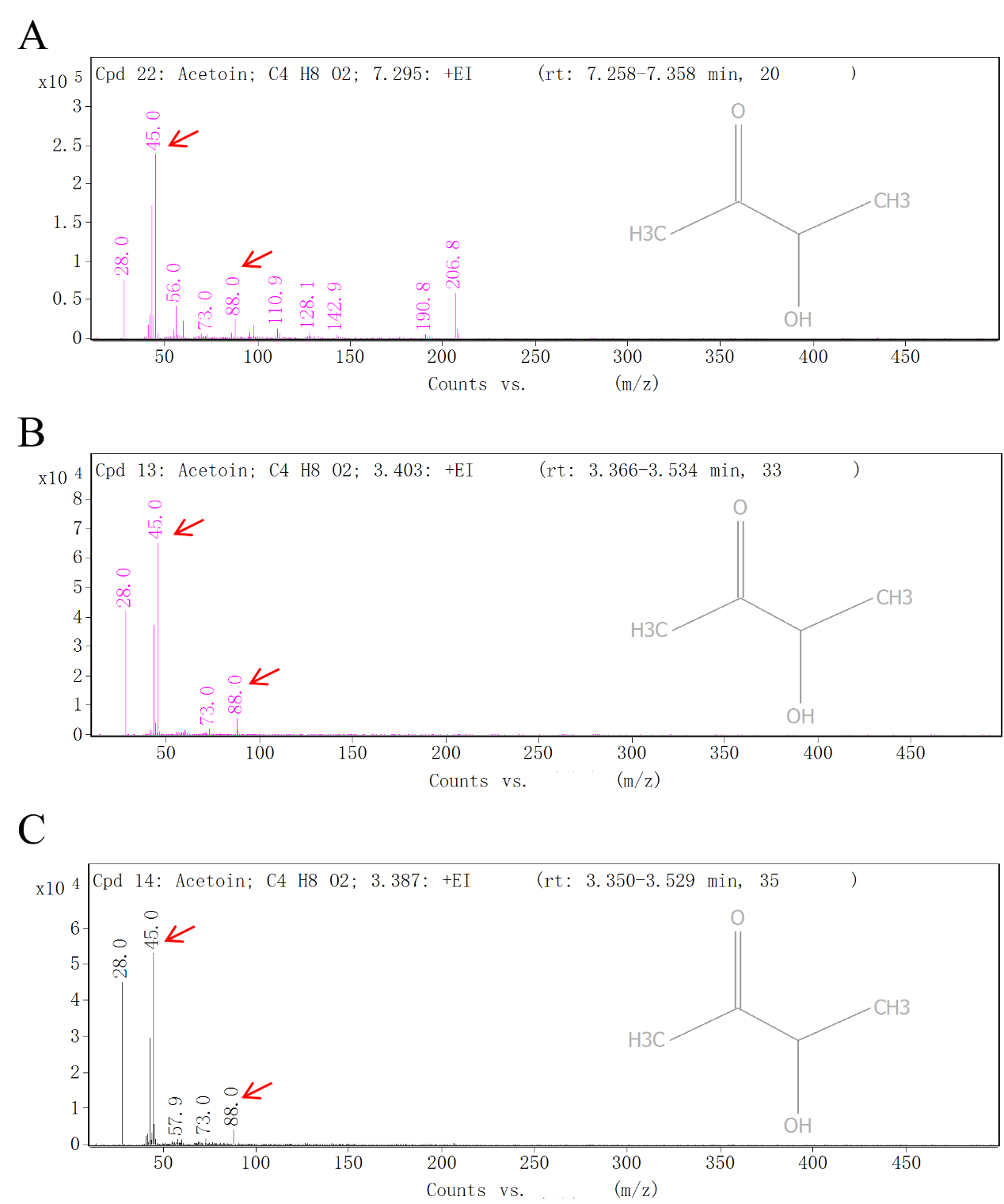


**Fig. S2.** GC-MS mass spectral comparison of acetoin standard (A), WT group (B), and Δ*Hfq* group (C).

Note: Red arrows indicate the diagnostic fragment ions (m/z 45.0 and 88.0) that confirm compound identity.


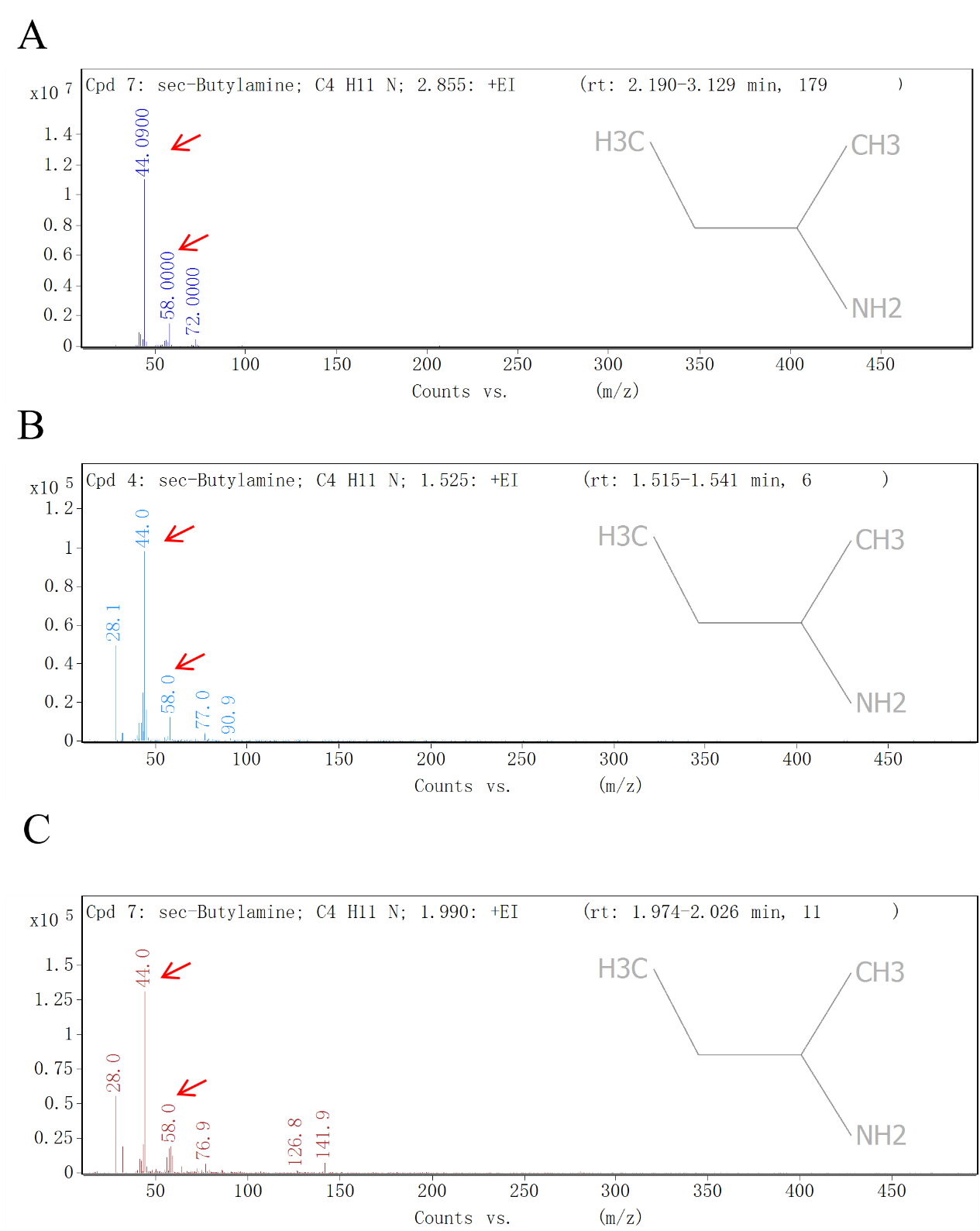


**Fig. S3.** GC-MS mass spectral comparison of sec-butylamine standard (A), WT group (B), and Δ*Hfq* group (C).

Note: Red arrows indicate the diagnostic fragment ions (m/z 44.0 and 58.0) that confirm compound identity.

**Construction process of the Δ*Hfq* strain**

**1.Construction of the targeting vector and preparation of the donor bacterium**

(1)Amplification and cloning of upstream and downstream homologous recombination arms

Table 1 PCR system.

| Component | Volume (μl) |
| --- | --- |
| bacterial broth | 0.2 |
| Hfq-5Fp/3F (50 pmol/μl) | 0.2 |
| Hfq-5R/3Rp (50 pmol/μl) | 0.2 |
| PrimeSTAR Max Premix (2×), TaKaRa | 10 |
| ddH_2_O | 9.4 |
| Total | 20 |

(2)Construction of targeting fragments using fusion PCR technology

Table 2 The reaction system of fusion PCR

| Component | Volume (μl) |
| --- | --- |
| Hfq-5F/5R PCR product | 4.7 |
| Hfq-3F/3R PCR product | 4.7 |
| Hfq-Linker (2 pmol/μl) | 0.2 |
| Hfq-5F (50 pmol/μl) | 0.2 |
| Hfq-3R (50 pmol/μl) | 0.2 |
| PrimeSTAR Max Premix (2×) | 10 |
| Total | 20 |


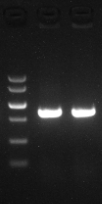


**M 2 1**

Fig. 1. Upstream homologous arm gene fragment and downstream homologous arm gene fragment

Note:M: DNA molecular weight standard. From top to bottom, the molecular weights are 2000 bp, 1500 bp, 1000 bp, 700 bp, 400 bp, and 200 bp successively, and the 1000 bp band is highlighted.

1: The amplification result of the upstream homologous recombination arm is 831 base pairs (bp).

2:The amplification result of the downstream homologous recombination arm is 831 base pairs (bp).


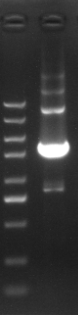


**M 1**

Fig. 2. Targeting sequence

Note:M: DNA molecular weight standard. From top to bottom, the molecular weights are 5000 bp, 3000 bp, 2000 bp, 1500 bp, 1000 bp, 750 bp, 500 bp, 250 bp, and 100 bp successively. Among them, the 750 bp band is highlighted.

1: Targeting sequence (upstream homologous recombination arm - downstream homologous recombination arm), with a length of 1662 base pairs (bp).

(3)Construction of the targeting vector：

Table 3 Restriction endonuclease digestion reaction

| Component | Volume (μl) |
| --- | --- |
| pCVD442Gm (~100 ng/μl) | 10 |
| 10×Tango buffer (MBI) | 5 |
| SmaI (10 U/μl,MBI) | 2 |
| ddH_2_O | 33 |
| Total | 50 |

Incubate the reaction at 30°C for 2 hours. After the restriction endonuclease digestion reaction is completed, add 3 μl of the dephosphorylating enzyme FastAP (MBI Fermentas) and incubate at 37°C for 30 minutes. After the reaction, the vector and the fusion PCR product are separated by 1% agarose gel electrophoresis respectively, purified by column centrifugation, and eluted in 50 μl of deionized water.

Table 4 Ligation Reaction System

| Component | Volume (μl) |
| --- | --- |
| pCVD442Gm/SmaI/FastAp (~50 ng/μl) | 2 |
| Hfq (~50 ng/μl) | 6 |
| 10×T4 buffer | 1 |
| T4 DNA ligase (5 U/μl,MBI) | 1 |
| Total | 10 |

After the ligation product was precipitated with isopropanol, it was washed with 70% ethanol and dissolved in 5 μl of deionized water. It was then transformed into *E. coli* DH5αλpir via electroporation (Micropulser, Bio - Rad). The transformed bacteria were cultured on LB plates (containing 40 μg/ml gentamicin) at 37°C until single colonies were formed.

Select one clone from the gentamicin - resistant plate. After sequencing to verify the correct sequence, conduct subsequent experiments and name it pCVD442Gm - Δ*Hfq*. Inoculate this clone into 3 ml of LB medium (containing 40 μg/ml gentamicin) and culture overnight at 37°C. The next day, purify the plasmid DNA by column centrifugation.

(4)Preparation of donor bacteria

The targeting vector pCVD442Gm - Δ*Hfq* was transformed into *E. coli* strain β2155 by electroporation. The transformed bacteria were spread on LB plates (containing 100 μg/ml ampicillin and 0.5 mM DAP) and cultured at 37°C until single colonies formed. This clone serves as the donor strain β2155/pCVD442Gm - Δ*Hfq* for the conjugation experiment.

**2.Conjugation experiment and screening of *Hfq* gene knockout strains**

(1)Streak and inoculate the recipient bacterium *Pantoea agglomerans* on a TSB plate, and culture it at 37°C until single colonies are formed. Pick a single colony and transfer it into 3 ml of TSB medium, and culture it at 37°C with a shaking speed of 220 rpm overnight.

(2)Pick a single colony of the recipient bacterium β2155/pCVD442Gm-Δ*Hfq* and inoculate it into 3 ml of TSB medium (containing 100 μg/ml of Ampicillin and 0.5 mM of Diaminopimelic acid, DAP). Culture it at 37°C with a shaking speed of 220 rpm overnight.

(3)Take 500 μl of the donor bacterium solution and 500 μl of the recipient bacterium solution, and mix them for the conjugation experiment.

(4)Take 50 μl of the bacterial solution after conjugation and spread it on an LB plate containing gentamicin (40 μg/ml), and culture it at 37°C until single colonies are formed.

(5)On the gentamicin-resistant plate, randomly select approximately 10 clones. Use a toothpick to pick each clone successively into the same tube containing 100 μl of LB medium. After mixing evenly, take 10 μl and inoculate it into 3 ml of TSB medium, and culture it at 37°C with a shaking speed of 220 rpm overnight. The next day, take 50 μl of the culture solution and spread it on an LB plate containing 10% sucrose (without NaCl and containing 10% sucrose), and incubate it at 37°C until single colonies are formed. Randomly select 14 single colonies and inoculate each into 3 ml of TSB medium respectively, and culture them at 37°C overnight. The next day, take a small amount of the bacterial solution and perform PCR identification using the internal primers of the *Hfq* gene. The results show that: except for clones No. 5, No. 7 and No. 11, all other clones have negative amplification, indicating that the *Hfq* gene in these clones should have been knocked out. However, further identification with outer primers is still required.

(6)Take a small amount of the bacterial solution of Clone No. 1 and perform a PCR reaction using outer primers. The results show that there is a specific amplification product with a length that meets the requirements. Send the PCR amplification product for sequencing verification. The sequencing results show that the *Hfq* gene has been knocked out, which is consistent with the design. The sequencing results are shown in the attachment. Confirm that this clone is an *Hfq* gene knockout clone, named Δ*Hfq*.

Table 5 PCR identification using internal primers system

| Component | Volume (μl) |
| --- | --- |
| bacterial broth | 0.5 |
| 10× Taq buffer | 5 |
| dNTP (2.5 mM) | 4 |
| Hfq-inF (50 pmol/μl) | 0.5 |
| Hfq-inR (50 pmol/μl) | 0.5 |
| Taq DNA polymerase (5 U/μl, MBI) | 0.5 |
| ddH_2_O | 39 |
| Total | 50 |


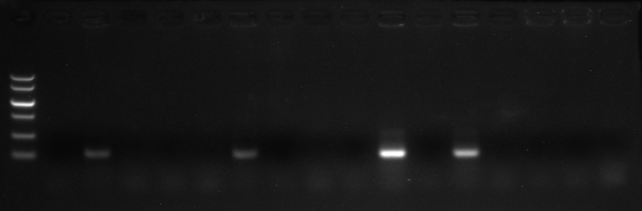


**M 16 15 14 13 12 11 10 9 8 7 6 5 4 3 2 1**

Fig. 3. The amplification result of the internal primer

Note:M: DNA molecular weight marker. From top to bottom, the molecular weights are successively: 2000, 1500, 1000, 700, 400, 200 bp, with 1000 bp being highlighted.

1-14: Amplification results of internal primers for Clone No. 1 to No. 14.

15: Amplification result of the internal primer of the original strain, and the length of the product is 227 bp.

16: Amplification result of the template-free negative control.

Table 6 PCR identification using outer primers system

| Component | Volume (μl) |
| --- | --- |
| bacterial broth | 0.5 |
| 10× Taq buffer | 5 |
| dNTP (2.5 mM) | 4 |
| Hfq-outF (50 pmol/μl) | 0.5 |
| Hfq-outR (50 pmol/μl) | 0.5 |
| Taq DNA polymerase (5 U/μl, MBI) | 0.5 |
| ddH_2_O | 39 |
| Total | 50 |


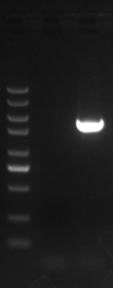


**M 2 1**

Fig. 4. The amplification result of the external primer

Note:M: DNA molecular weight marker. From top to bottom, the molecular weights are successively: 5000, 3000, 2000, 1500, 1000, 750, 500, 250, 100 bp, among which 750 bp is highlighted.

1: Amplification result of the outer primers of Clone No. 1, with a length of 1786 bp.

2: Amplification result of the template-free negative control.
